# Supplementary material for: Chronic loss of inhibition in piriform cortex following brief, daily optogenetic stimulation
Source: Cell Rep. Author manuscript; Available in PMC 2021 May 6. (PMC8102022; doi:10.1016/j.celrep.2021.109001)
Supplement: 1 [file NIHMS1696727-supplement-1.pdf]

Cell Reports, Volume *35*

Supplemental information

Chronic loss of inhibition in piriform cortex following brief, daily optogenetic stimulation

Brendan Ryu, Shivathmihai Nagappan, Fernando Santos-Valencia, Psyche Lee, Erica Rodriguez, Meredith Lackie, Jun Takatoh, and Kevin M. Franks

Supplementary Materials for Ryu et al, 2020

Figure S1. related to Figure 1.

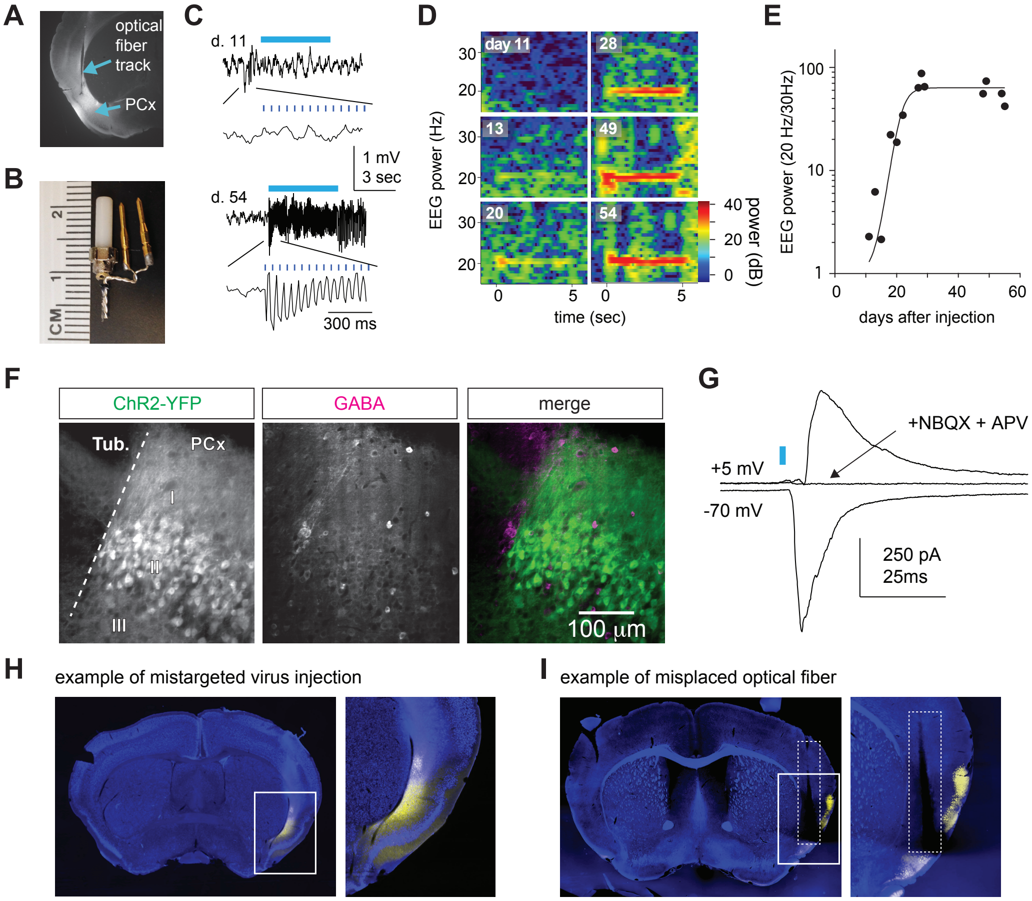

Figure S1. Timeline and specificity of ChR2 expression (**A**) Coronal brain section showing ChR2-YFP expression and track from optical fiber. This animal was implanted with only an optical fiber-coupled ferrule. Tissue damage was considerably more extensive when using optrodes. (**B**) Example optrode. (**C**) Example LFPs in response to brief light pulses at 20 Hz, recorded in the same mouse 11days (*top*) and 54 days (*bottom*) after virus injection. The same light intensity was used for all stimuli. Insets show time of stimulus onset at expanded time scales. (**D**) Spectrograms in response to 20 Hz stimuli at different times after virus injection from the same mouse, including the examples shown in C. (**E**) Evolution of ChR2 expression measured as power of the LFP at 20 Hz normalized by the power of the LFP at 30 Hz during stimulation. As ChR2 expression increases the LFP becomes increasingly stimulus-locked until it plateaus 3 weeks after virus injection. Solid line is a sigmoidal fit to the data. (**F**) ChR2-YFP expression at the boundary of the PCx, in which most cells are glutamatergic, and the olfactory tubercle, which is predominantly GABAergic. Note the abrupt termination of ChR2-YFP expression at the PCx-tubercle boundary. Note also that the few GABA-positive neurons in PCx do not express ChR2-YFP. Scale bar is 100 µm. (**G**) Example whole-cell voltage-clamp recording from an uninfected neuron in response to a brief (2 ms) light pulse (blue bar). Light evoked strong inward currents (i.e. EPSCs) at V_m = -70 mV and outward currents (IPSCs) at V_m = +5 mV. IPSCs were completely blocked by glutamate receptor antagonists (NBQX, 10 µM; D-APV 50 µM), consistent with disynaptic feedback

inhibition with ChR2 expression being selective for glutamatergic and not GABAergic neurons. IPSCs were also blocked by gabazine (10 µM, not shown). (**H**) Example of an experiment in which virus injection was mistargeted so that there was negligible ChR2-YFP expression in PCx. Panel on right shows expanded region in box on left. (**I**) Example of an experiment in the optical fiber was lowered through PCx. Panel on right shows expanded region in box on left. Dashed line demarcates fiber tract.

Figure S2. related to Figure 1.

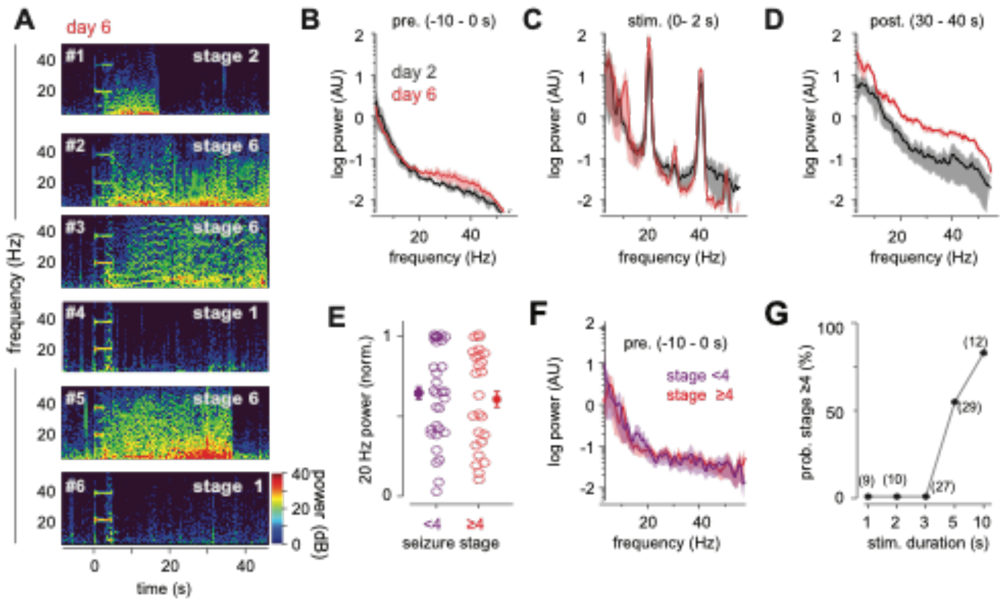

Figure S2. Strong seizures are only evoked on a subset of trials. (**A**) Example spectrograms for all stimuli on day 6 from a representative OpK animal. Stimulus number is indicated in top left. Note that stimuli 1, 4 and 6 fail to evoke seizures. (**B**) Average LFP power spectra during the 10 s before stimulus onset, averaged across all trials from animals with implanted electrodes (n = 5 mice) on day 2 (black trace) and day 6 (red trace). (**C**) As in B, but for the first 2 s of the 20 Hz stimulus train. (**D**) As in B, but for 10 s. starting 25 s after the stimulus ended. (**E**) The was no difference in stimulus strength on trials that did or did not evoke seizures in OpK mice. Graph shows power of LFPs at 20 Hz during first 2 s of all 6 stimuli on days 5 and 6. Trials are sorted by stimuli that unsuccessfully (purple circles) or successfully (red circles) evoked a stage ≥4 seizure on each day, and normalized by the maximum power at 20 Hz recorded on each day, regardless of whether that trial was successful (mean ± s.e.m.: seizure trials, 0.65 ± 0.053, n = 32 trials from 5 mice; no seizure trials, 0.61 ± 0.061, n = 25 trials from 5 mice; $p = 0.53$, Mann-Whitney U test). (**F**) Average power spectra of LFPs before stimulus onset for all trials on days 5 and 6 sorted by trials that did (red trace) and did not (purple trace) evoke stage ≥4 seizures. We found no obvious differences in LFPs before stimulation in trials that did and did not evoke seizures. (**G**) Probability of evoking a stage ≥4 seizure in already optokindled mice (i.e. beyond the first 6 days) as a function of stimulation duration. Each day a single 5 s stimulus was presented first and was not included in this analysis. Numbers above each point indicate number of trials for each duration.

Figure S3, related to Figure 1.

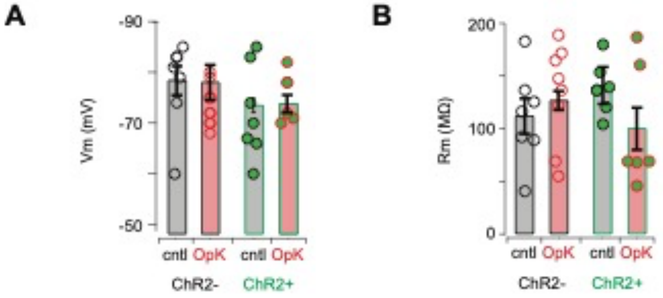

Figure S3. No change in intrinsic excitability or EPSC paired-pulse ratios. (**A**) Resting membrane potentials (V_m) were equivalent in ChR2-positive (green outlines) and uninfected cells (black outlines) from both OpK (red shading) or control (grey shading) animals. Two-way ANOVA found no significant effects for ChR2 expression ($F_{1,24}$=.0.447, p=0.510), optokindling ($F_{1,24}$=.0.121, p=0.731), or an interaction $F_{1,24}$=.2.16, p=0.154). (**B**) As in A, but for input resistance. Two-way ANOVA found no significant effects for ChR2 expression ($F_{1,24}$=.0.242, p=0.627), optokindling ($F_{1,24}$=.0.036, p=0.852), or an interaction $F_{1,24}$=.2.94, p=0.010).

Figure S4, related to Figure 3.

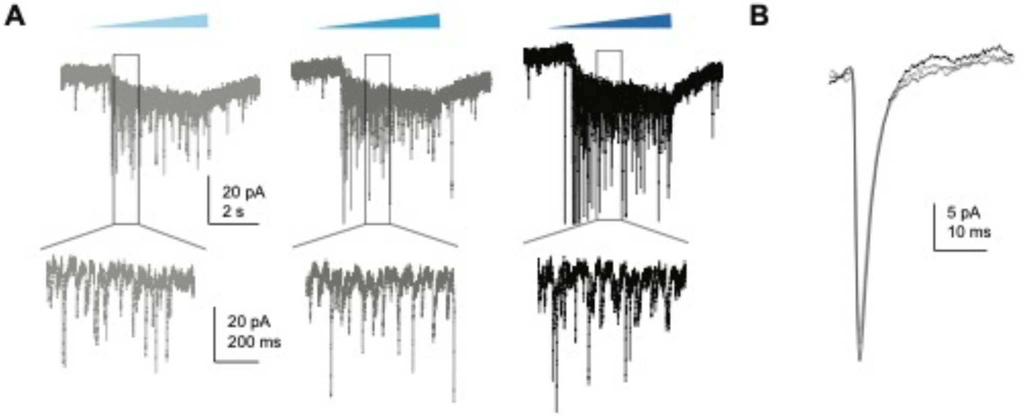

Figure S4. Light intensity changes uEPSC frequency but not amplitude. (**A**) Three traces from an example recording in which uEPSCs were evoked by ramping 5-second light pulses at increasing intensities (blue triangels). Traces below correspond to insets indicated by the boxes on traces above. (**B**) Overlaid uEPSCs averaged over at least 100 well-isolated individual events recorded at each of the three stimulus intensities.

Figure S5, related to Figure 5

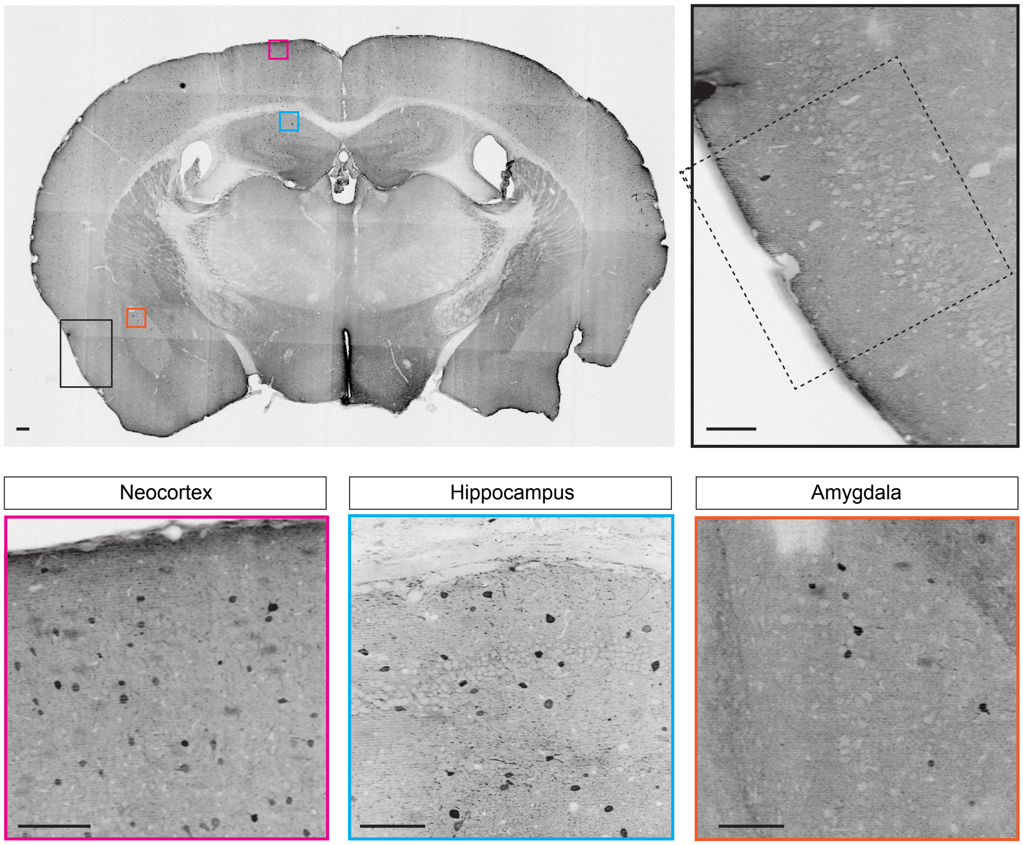

Figure S5. Robust GABA expression outside of PCx.
Coronal section from an OpK mouse stained for GABA. Insets show enlarged sections from PCx (*right*), and ipsilateral neocortex, hippocampus and amygdala. Note that GABA staining is depleted in PCx but robust elsewhere. Dashed box over PCx insert indicates the 500 µm x 500 µm window that was aligned to the top of layer I and used for quantification. All scale bars are 100 µm. All sections were harvested 24 hours after the sixth purple trace stimulation.

Figure S6, related to Figure 6.

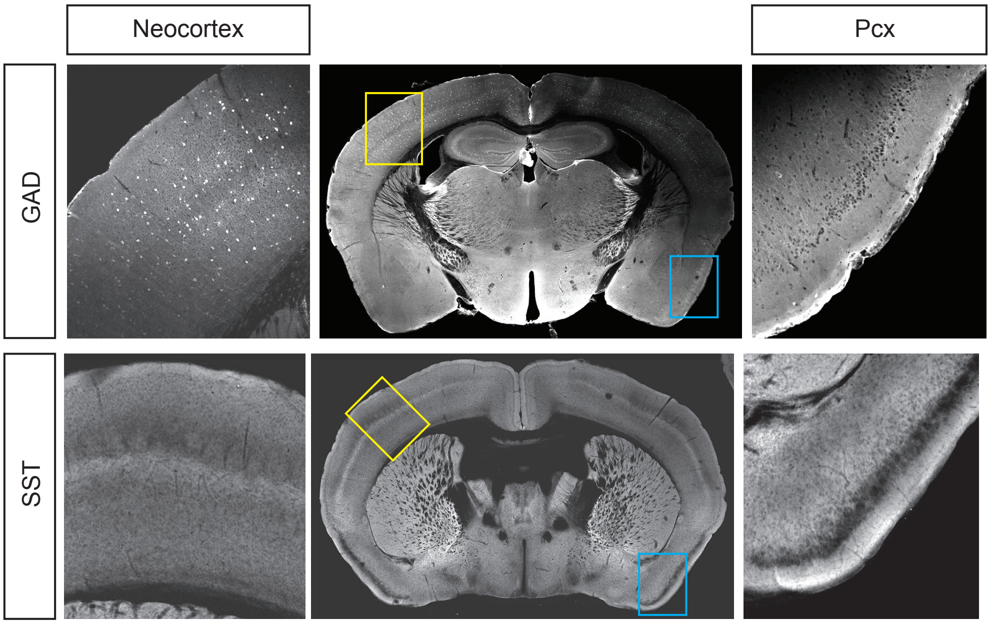

Figure S6. Failed labeling of PCx interneurons with GAD and SSt antibodies.
Example of whole-brain coronal sections stained using either GAD (*top*, rabbit anti-GAD2, 1:500, Cell Signaling Technology 5843S) or SSt antibodies (*bottom*, rabbit anti-SST, 1:500, Invitrogen 701935). In all cases we failed to label interneuron somata in PCx. Panels on left and right show magnified images of the neocortex (yellow box) and pPCx (blue box) on the main panel. Note, for example, that we could clearly label neocortical GAD-positive neurons in neocortex but not in pPCx in the same section.
