# Supplementary material for: Chronic loss of inhibition in piriform cortex following brief, daily optogenetic stimulation
Source: Cell Rep. Author manuscript; Available in PMC 2021 May 6. (PMC8102022; doi:10.1016/j.celrep.2021.109001)
Supplement: 4 [file NIHMS1696727-supplement-4.pdf]

Cell Reports

Article

Chronic loss of inhibition in piriform cortex following brief, daily optogenetic stimulation

Graphical abstract

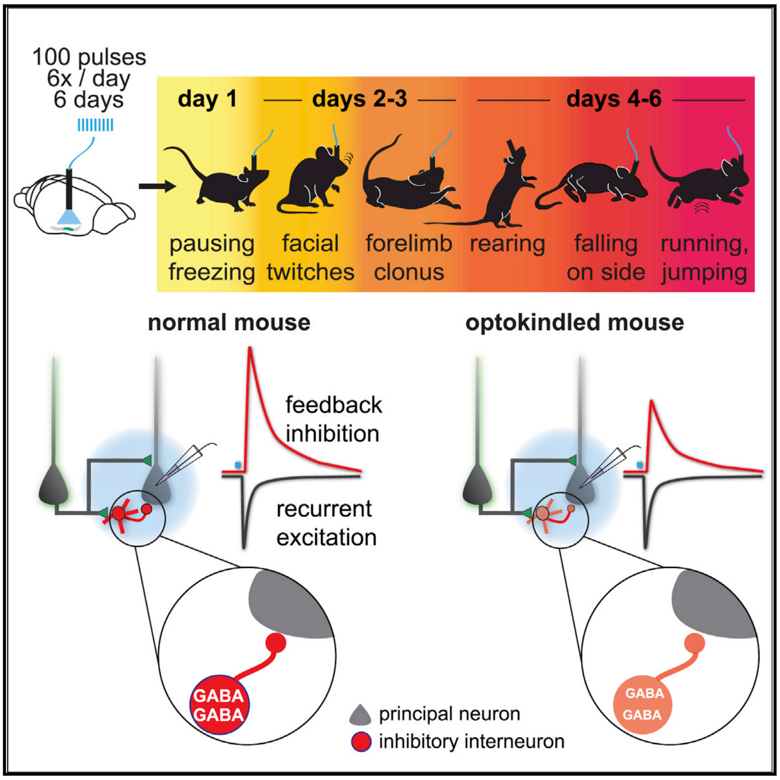

Authors

Brendan Ryu, Shivathmihai Nagappan, Fernando Santos-Valencia, ..., Meredith Lackie, Jun Takatoh, Kevin M. Franks

Correspondence

franks@neuro.duke.edu

In brief

Ryu et al. use optogenetics to briefly activate principal neurons in mouse piriform cortex. After 4 days, previously innocuous stimuli evoke massive, generalized seizures. “Optokindling” does not strengthen recurrent excitation; instead, it weakens feedback inhibition by decreasing synaptic cleft GABA concentrations and slowing vesicle refilling, consistent with decreased GABA synthesis.

Highlights

- Brief, daily optogenetic stimulation rapidly induces kindling epileptogenesis
- Highly recurrent, allocortical areas are particularly susceptible to “optokindling”
- Selectively activating principal cells does not strengthen recurrent excitation
- Instead, “optokindling” weakens feedback inhibition by disrupting GABA synthesis

Ryu et al., 2021, Cell Reports *35*, 109001
April 20, 2021 © 2021 The Author(s).
https://doi.org/10.1016/j.celrep.2021.109001

CellPress

Cell Reports

CellPress
OPEN ACCESS

Article

Chronic loss of inhibition in piriform cortex following brief, daily optogenetic stimulation

Brendan Ryu,[1] Shivathmihai Nagappan,[1] Fernando Santos-Valencia,[1] Psyche Lee,[1] Erica Rodriguez,[1] Meredith Lackie,[1] Jun Takatoh,[1] and Kevin M. Franks[1,2,*]

[1]Department of Neurobiology, Duke University Medical School, Durham, NC 27705, USA
[2]Lead contact
*Correspondence: franks@neuro.duke.edu
https://doi.org/10.1016/j.celrep.2021.109001

SUMMARY

It is well established that seizures beget seizures, yet the cellular processes that underlie progressive epileptogenesis remain unclear. Here, we use optogenetics to briefly activate targeted populations of mouse piriform cortex (PCx) principal neurons *in vivo*. After just 3 or 4 days of stimulation, previously subconvulsive stimuli trigger massive, generalized seizures. Highly recurrent allocortices are especially prone to "optokindling." Optokindling upsets the balance of recurrent excitation and feedback inhibition. To understand how this balance is disrupted, we then selectively reactivate the same neurons *in vitro*. Surprisingly, we find no evidence of heterosynaptic potentiation; instead, we observe a marked, pathway-specific decrease in feedback inhibition. We find no loss of inhibitory interneurons; rather, decreased GABA synthesis in feedback inhibitory neurons appears to underlie weakened inhibition. Optokindling will allow precise identification of the molecular processes by which brain activity patterns can progressively and pathologically disrupt the balance of cortical excitation and inhibition.

INTRODUCTION

Cortical neurons communicate via recurrent excitatory synaptic connections, with each cell typically receiving excitatory inputs from thousands of other cortical neurons (Braitenberg and Schüz, 1998; Douglas et al., 1995). Ongoing activity *in vivo* produces fluctuating levels of excitatory input that must be dynamically balanced by recurrent inhibition (Borg-Graham et al., 1998; Haider et al., 2006; Shadlen and Newsome, 1998; Shu et al., 2003; van Vreeswijk and Sompolinsky, 1996), and disruptions in this excitation/inhibition (E/I) balance can lead to hyperexcitability and seizures.

Clinical observations have long implicated seizures themselves as causal factors in the development and/or worsening of epilepsy in multiple syndromes (Gowers, 1881). This hypothesis has been supported by kindling epileptogenesis, in which repeated initiation of focal and initially subconvulsive seizures causes progressively more severe general seizures (Goddard, 1967). Kindling, therefore, provides an opportunity to elucidate the cellular and molecular processes that underlie epileptogenesis. Yet, despite extensive study, how seizures and seizure-like activity promote epileptogenesis remains unclear. Candidate mechanisms for kindling epileptogenesis include changes in intrinsic excitability, increases in the strength or number of excitatory connections, death of inhibitory interneurons, decreases in the strength or number of inhibitory connections, changes in chloride reversal potential that weaken synaptic inhibition, and others (McNamara, 1995; Staley, 2015).

Two major technical limitations have precluded definitive identification of the cellular and molecular processes that underlie kindling. First, electrical stimulation activates cells non-selectively, including axons of passage, complicating interpretation of which cells' activity directly mediates epileptogenesis. Second, cells activated electrically *in vivo* cannot be selectively reactivated *in vitro*, and this has confounded attempts to probe underlying cellular mechanisms. However, the development of optogenetic tools overcomes these limitations by allowing targeted activation of specific populations of neurons *in vivo* and their selective reactivation *in vitro*, thereby obviating both these problems. Recently, an optogenetic kindling (OpK) model was described and carefully characterized, validating that this approach bears the hallmarks of classical kindling models but with the specificity and precision of optogenetic stimulation (Cela et al., 2019).

Neural plasticity associated with kindling has long been thought to represent an extreme instantiation of the same principles and characteristics of plasticity associated with learning and memory (Goddard and Douglas, 1975). Consistent with this view, the piriform cortex (PCx), a plastic allocortical structure that is presumed to be the locus for odor learning and memory (Wilson and Sullivan, 2011), is a highly epileptogenic circuit in rodents that is especially susceptible to kindling epileptogenesis (Löscher and Ebert, 1996). PCx has also been implicated in seizures in humans (Vaughan and Jackson, 2014).

The PCx is a three-layered allocortex in which principal cells receive excitatory afferent inputs from the olfactory bulb on their distal apical dendrites. These cells form a recurrent circuit by

This is an open access article under the CC BY license (http://creativecommons.org/licenses/by/4.0/).

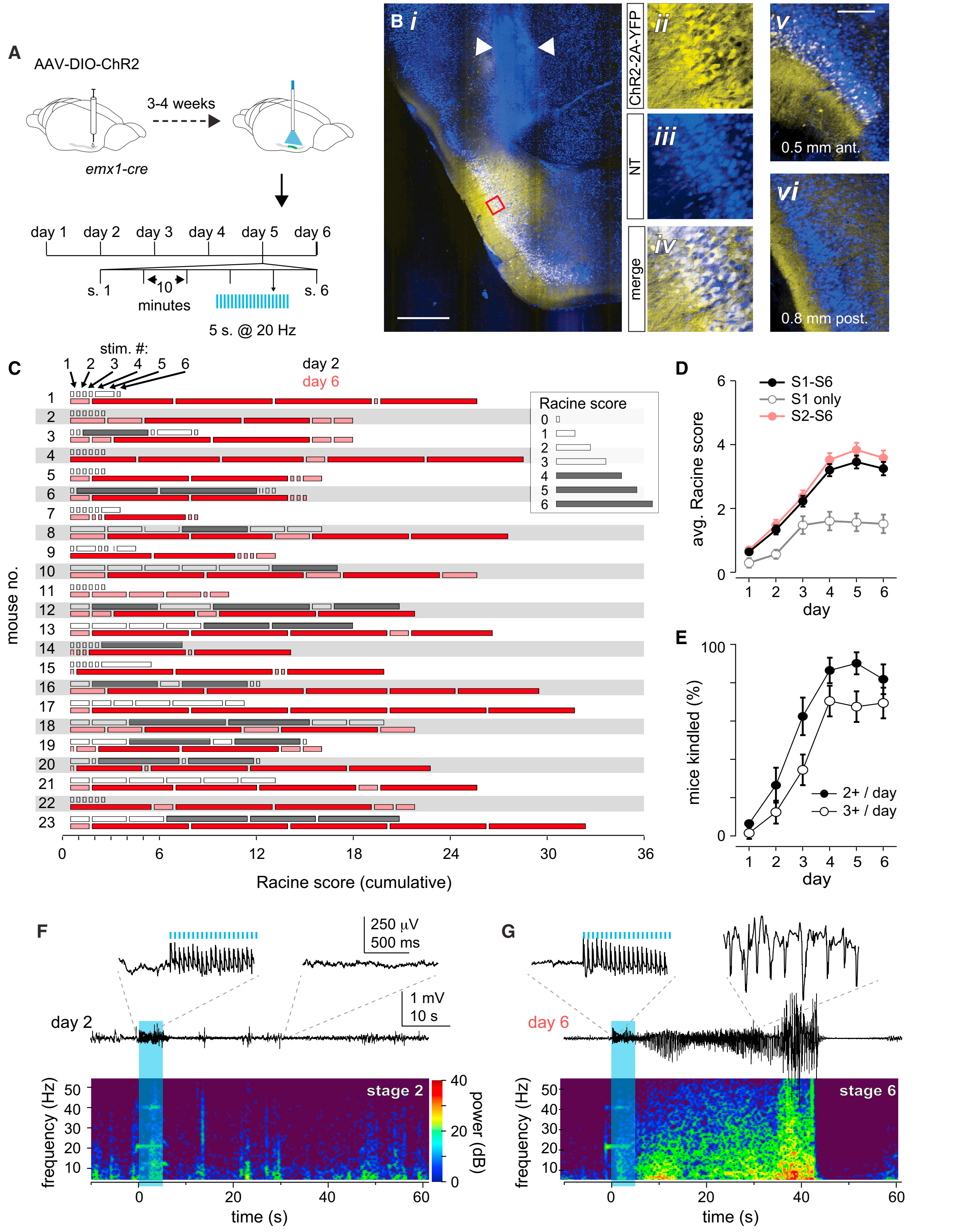

(legend on next page)

extending their axons across PCx, forming long-range excitatory connections onto other PCx pyramidal cells (Choy et al., 2017; Franks et al., 2011; Hagiwara et al., 2012; Johnson et al., 2000). Odors activate ensembles of neurons distributed across PCx (Rennaker et al., 2007; Roland et al., 2017; Stettler and Axel, 2009); however, only a fraction of responsive PCx cells are thought to be directly activated by olfactory bulb input: many odor-responsive cells are likely driven by subthreshold bulb input that is augmented by recurrent excitation (Davison and Ehlers, 2011; Franks et al., 2011; Poo and Isaacson, 2011; Stern et al., 2018). Broadly, there are two classes of GABAergic inhibitory neurons in PCx that temper this excitation. First, GABAergic interneurons located in layer I receive excitatory input from olfactory bulb mitral cells and provide feedforward inhibition onto PCx principal neurons. Second, GABAergic interneurons located in layers II and III receive excitatory inputs from PCx principal neurons and then provide feedback inhibition back onto the principal cells (Franks et al., 2011; Large et al., 2016; Stokes and Isaacson, 2010; Suzuki and Bekkers, 2012), which checks and terminates odor-driven PCx activity (Bolding and Franks, 2018).

According to theoretical models, strengthening excitatory synaptic connections between neurons can produce odor-specific cortical cell assemblies that stabilize and reinforce PCx odor ensembles (Haberly, 2001; Wilson and Sullivan, 2011). The long-range recurrent network provides a substrate for interconnecting these neurons, and recent *in vivo* experiments provide indirect evidence for this model (Bolding et al., 2020; Pashkovski et al., 2020). Consistent with these models, *in vitro* experiments have shown that recurrent synapses between PCx neurons exhibit robust long-term potentiation (LTP) that persists into adulthood (Jung et al., 1990; Kanter and Haberly, 1990; Poo and Isaacson, 2007). Whether such augmentation occurs *in vivo* and, if so, whether and how this plasticity alters the E/I balance is not known.

We therefore took advantage of its simple laminar organization and the specificity afforded by optogenetics to repeatedly activate a subset of mouse PCx principal neurons *in vivo* until brief light stimulation evoked generalized seizures. We then selectively reactivated these cells *in vitro* to examine how their repeated activation altered the E/I balance in this recurrent and plastic cortical circuit.

RESULTS

Optokindling

We examined the effect of briefly activating a subset of PCx principal neurons *in vivo* each day for 6 days (Figure 1A). To do this, we injected Cre-dependent adeno-associated viruses (AAVs) into anterior PCx (aPCx) of Emx1-Cre mice to selectively express Channelrhodopsin-2 (ChR2) in a focal subset of PCx principal cells (Figures 1B and S1A). We infected $\sim 10^5$ cells, or about $\sim$10% of ipsilateral PCx (Srinivasan and Stevens, 2018). We implanted a ferrule-coupled optical fiber just above PCx to activate injected cells. In some cases, the optical fiber was coupled with a bipolar electrode to measure local field potentials (Figure S1B). In initial experiments, we waited at least 4 weeks after virus injections to ensure that any changes we observed following daily stimulation were not a consequence of increasing ChR2 expression levels (Figures S1D and S1E). We also verified that ChR2 was expressed in glutamatergic and not GABAergic neurons (Figures S1F and S1G).

We placed mice in an empty arena and briefly activated ChR2-positive ($ChR2^+$) neurons with trains of light pulses (100 × 10-ms pulses at 20 Hz; Figure 1A), which were presented six times per day, 10 min apart. We repeated this protocol for 6 consecutive days. Laser power was titrated during stimulation on the first day (range: 0.5–2.0 mW) so that mice paused briefly during the light train (i.e., Racine scale 0; see STAR Methods) but did not exhibit orofacial twitching (i.e., Racine scale 1), and then immediately continued behaving as before as soon as the stimulus ended (day 1 Racine score: mean ± SD, 0.64 ± 1.02; n = 138 = 23 mice × 6 stimuli/day; Figures 1C and 1D). The same laser power was then used for all subsequent stimuli in that animal. On day 2, some mice exhibited mild focal seizures in response to some of the light stimuli (day 2: mean Racine score ± SD, 1.33 ± 1.48; Video S1). However, from the fourth day onward, mice reliably exhibited multiple generalized (stage 4 or higher) seizures per day (day 6 Racine score: mean ± SD, 3.25 ± 2.48; Figure 1D; Video S2). Seizures began during or shortly after the light stimulation and typically persisted until 30–40 s after the termination of the stimulus. Mice were deemed to be successfully "optokindled" (OpK) if they exhibited stage $\geq$ 4 seizures on at least three of six stimuli on any one day (22/23 mice; Figure 1E). We

Figure 1. Robust, prolonged seizures develop following daily optical stimulation

(A) Experimental schematic. Cre-dependent AAVs conditionally expressing ChR2-YFP were injected into aPCx of Emx1-Cre mice. After waiting for viral expression to stabilize, infected cells were briefly photoactivated with trains consisting of 100 pulses (10 ms) at 20 Hz. Each train was presented six times per day (S1–S6), 10 min apart. This protocol was repeated for 6 days.

(Bi) Example ChR2-YFP expression pattern in aPCx. Arrowheads indicate scarring from the optical fiber track.

(Bii–Biv) Higher magnification of insets in red box in (i). ChR2-YFP expression is focal, with few $ChR2\text{-}YFP^+$ cells rostral (v) and no $ChR2\text{-}YFP^+$ cells caudal (vi) to the injection site. Scale bars: 500 μm (i); 250 μm (v).

(C) Seizure severity quantified as modified Racine scores for each of the six stimuli presented on days 2 (black bars) and 6 (red bars). Bar size corresponds to Racine seizure score. Data are shown for the first cohort of 23 mice in which the optokindling protocol was followed most strictly.

(D) Mean daily Racine scores averaged over across all six daily stimuli (closed circles). Note that the first stimulus of the day (open circles) was less effective at evoking seizures than the subsequent five stimuli (red circles). Error bars indicate SEM.

(E) Probability of kindling expressed as fraction of mice that expressed at least two (closed circles) or three (open circles) stage $\geq$ 4 seizures on any of the six stimuli presented on each day.

(F) Representative day 2 LFP recording (top) and spectrogram (bottom). The blue shading indicates the timing of the 5-s-long, 20-Hz light train. The insets provide expanded views during the stimulation period and approximately 30 s later.

(G) Example LFP recording on day 6 from same mouse as (F). Note that the seizure persists and intensifies long after the stimulus ends and then terminates abruptly. The main trace, the inset, and the spectrogram are all drawn the same scale as those in (F).

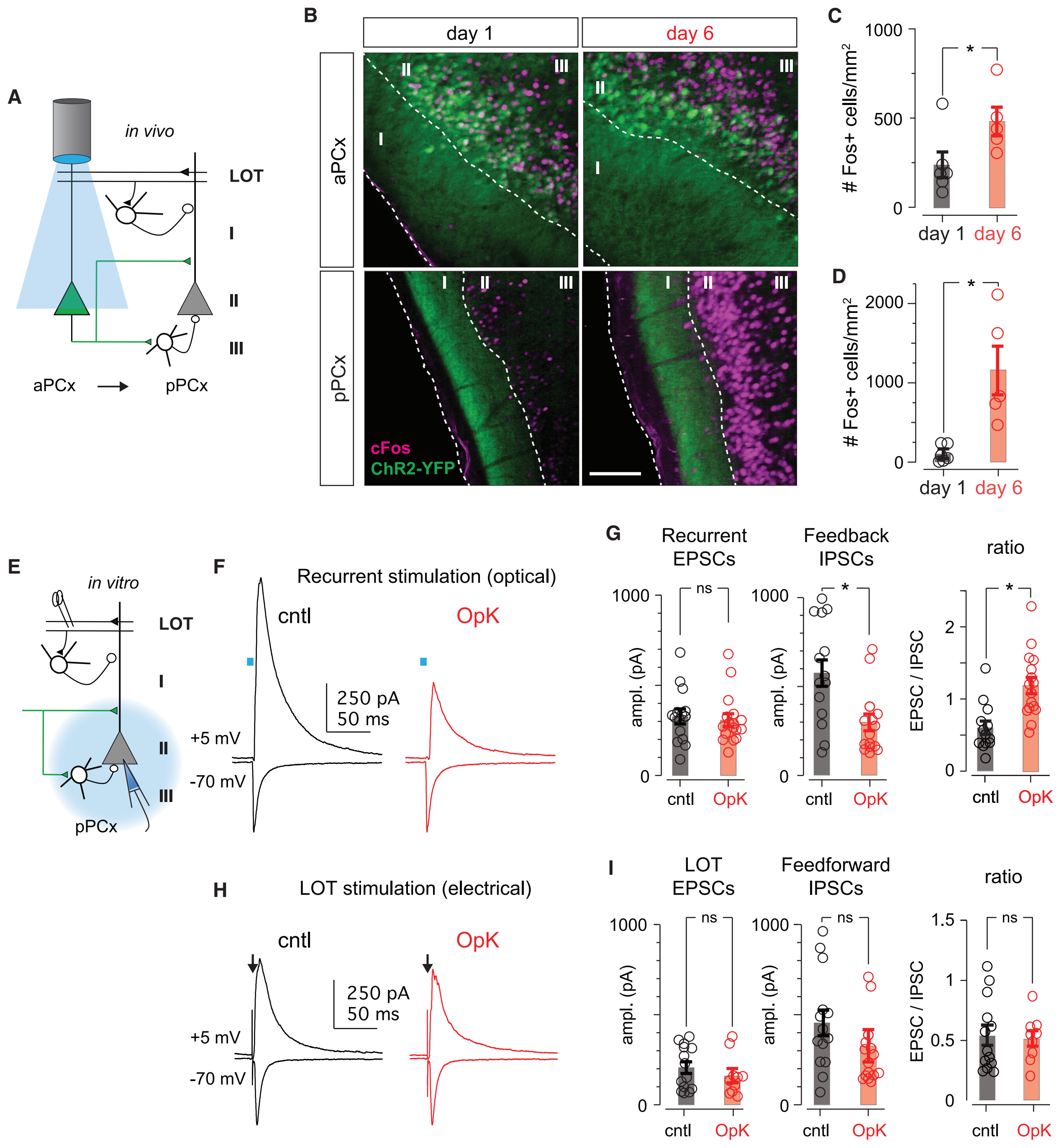

Figure 2. Optokindling disrupts the balance of recurrent excitation and feedback inhibition

(A) Schematic showing *in vivo* stimulation paradigm. ChR2$^+$ cells in aPCx are activated optically. ChR2$^-$ cells in pPCx receive excitatory recurrent inputs from aPCx but are not directly activated.

(B) ChR2-YFP and *Fos* expression in aPCx and pPCx from mice killed 1 h after the last stimulus on either day 1 or 6. Dashed white lines demarcate layers, which are labeled by numerals. ChR2-YFP$^+$ cells in aPCx extend axons through layer I of pPCx. Scale bar: 100 μm.

(C) Number of *Fos*$^+$ neurons in aPCx after the first or sixth day of stimulation. Bars indicate mean *Fos*$^+$ cell counts across animals, and error bars represent SEM; each circle represents the mean number of *Fos*$^+$ neurons per mouse averaged across four sequential 50-μm sections.

(D) As in (C) but for pPCx.

(E) Schematic of *in vitro* slice experiments. We recorded from ChR2$^-$ pyramidal cells and either measured monosynaptic recurrent EPSCs and disynaptic feedback IPSCs evoked by optically activating ChR2$^+$ axons. We also activated excitatory olfactory bulb inputs and disynaptic feedforward inhibition by electrically stimulating mitral cell axons in the LOT.

(legend continued on next page)

never observed spontaneous seizures between bouts of optical stimulation. A small subset of mice never exhibited seizures, and invariably, this corresponded with either a mistargeting of the virus injection and minimal ChR2 expression in PCx (Figure S1H) and/or mistargeting of the optical fiber (Figure S1I), and these mice were not included in these analyses.

To examine the neural activity underlying these seizures, we recorded LFPs in a subset of mice before, during, and after optical stimulation. LFP power at 20 Hz, and its harmonics, increased markedly during optical stimulation. Importantly, LFPs before and during stimulation were indistinguishable on day 2 versus day 6 (Figures 1F and 1G; Figure S2C). On trials in which light did not induce seizures, LFPs returned to baseline levels immediately after stimulation (Figure 1F). However, we observed strong, stimulus-evoked electrographic seizures, characterized by a dramatic increase in broad-frequency activity, that corresponded to the behavioral seizures (Figure 1G). Like behavioral seizures, LFP activity evolved slowly and typically manifested after the end of the 5-s stimulus, becoming progressively more intense over tens of seconds, peaking ~30 s after stimulus offset, and then ending abruptly (mean duration ± SD after stimulus onset: 45 ± 17 s, n = 37 stage ≥ 4 seizures on days 4–6, n = 5 mice; Figure S2A). Note, therefore, that the seizures were not maintained by the external activation of $ChR2^+$ neurons; instead, photostimulation initiated regenerative cascades of neural activity that sustained and increased in intensity long after the 5-s stimulus had ended (Figures 1G and S2A).

Different timescales for plasticity

In addition to the changes in LFPs and seizure severity that evolved over several days, we also observed plasticity that occurred on shorter timescales. For example, although kindled mice exhibited multiple "strong" (i.e., stage ≥ 4) seizures per day, the first stimulus on any given day rarely evoked seizures (only 15 stage ≥ 4 seizures from 138 first stimuli; i.e., 23 mice × 6 days). Moreover, even once kindled (i.e., on days 4, 5, and 6), mice often exhibited two successive strong seizures followed by a complete failure to evoke a seizure with the next stimulus (Figures 1C and S2A). We could not predict why some stimuli evoked seizures, whereas others did not (Figures S2B–S2D). For example, although there was considerable trial-to-trial variability in the power of the LFP at 20 Hz during stimulation (coefficient of variation = 0.80 across stimuli), there was no systematic difference in the strength of stimuli that did or did not evoke seizures (Figure S2E). Moreover, we found no obvious differences in LFPs immediately preceding stimulation that were predictive of whether or not a seizure would occur (Figure S2F). We also observed plasticity on a timescale of seconds. Specifically, we presented already-kindled mice (i.e., after 6 days of stimulation) with an initial 5-s stimulus each day and then varied the length of subsequent stimuli. Subsequent 5-s stimuli evoked strong seizures on 55% of trials. However, we never evoked seizures with 1-, 2-, or 3-s stimuli. By contrast, we evoked strong seizures on 83% of trials using 10-s stimuli (Figure S2G). Synaptic plasticity on the timescale of tens to hundreds of milliseconds is also likely to be important for network excitability. Although we have not attempted to do so here, OpK provides a method to directly probe how plasticity at these various timescales is affected. Here, we focused on revealing the changes in circuitry that underlie the progressive, days-long evolution in responses to repeated stimulation.

Activity spreads through a disruption in the balance of recurrent excitation and feedback inhibition

We next examined the pattern and spread of activity within PCx following OpK using the expression of the immediate-early gene, *Fos*, as a proxy for neural activity. We compared Fos expression following 1 or 6 days of stimulation in both aPCx, the site of ChR2 expression and photoactivation, and 1–2 mm downstream, in posterior PCx (pPCx; Figure 2A). Following only 1 day of stimulation, we observed high levels of Fos expression in aPCX, where many Fos^+ cells were also $ChR2^+$ (Figure 2B). Although these neurons send excitatory projections to pPCx, we observed many fewer Fos^+ cells in pPCx (day 1 aPCx, 238 ± 71.8 cells/mm^2; day 1 pPCx, 103 ± 43.4 cells/mm^2, n = 6 mice; p = 0.046, signed-rank test; Figure 2C), indicating that stimulation of aPCx neurons initially does not robustly activate downstream pPCx neurons. More Fos^+ cells were found in aPCX after day 6 than day 1 (day 6 aPCx, 481 ± 79.8 cells/mm^2; n = 5 mice; p = 0.0494, unpaired t test), with comparatively more Fos^+ cells in deeper layer 2 and layer 3 after the sixth day. Strikingly, however, we now observed robust and extensive Fos expression in pPCx (day 6 pPCx, 1,155 ± 308 cells/mm^2; n = 5 mice; p = 0.031, signed-rank test; Figures 2B and 2D) and, notably, many more Fos^+ cells in pPCx after 6 days versus 1 day of stimulation (p = 0.0262, unpaired t test). These data are consistent with a model in which OpK disrupts the balance of recurrent excitation and feedback inhibition, so that after OpK, recurrent excitatory input from $ChR2^+$ cells in aPCx effectively recruits uninfected pPCx neurons whose activity was initially held in check by strong feedback inhibition.

To test this model, we isolated acute brain slices from OpK animals immediately after the sixth day of *in vivo* stimulation (day 6) or from control (cntl) mice that had been injected with AAVs expressing ChR2 and fiber implanted, but not stimulated. We obtained whole-cell voltage-clamp recordings from uninfected layer II pyramidal cells caudal to the infection site (i.e., caudal aPCx through rostral pPCx) to examine the synaptic responses evoked by activating the axon terminals of $ChR2^+$ cells (Figure 2E). The ratios of monosynaptic excitatory postsynaptic currents (EPSCs) and disynaptic inhibitory synaptic currents (IPSCs) evoked by activating the same set of $ChR2^+$ presynaptic inputs

(F) Recurrent EPSCs (V_m = −70 mV) and feedback IPSCs (V_m = +5 mV) in an example cell from an unstimulated control (cntl) mouse (black traces) and in a cell from an OpK mouse (red traces) evoked by 2-ms light pulses (blue bars).

(G) Amplitudes of recurrent EPSCs (left), disynaptic feedback IPSCs (middle), and their ratios (right) evoked using equivalent stimuli in cells from cntl and OpK mice. Each circle represents the amplitude or amplitude ratio for a single cell, bars indicate average across cells, and error bars represent SEM.

(H) Example afferent EPSCs and feedforward IPSCs evoked by electrically stimulating the LOT (arrows). Stimulus artifacts have been truncated for clarity.

(I) Summary of evoked EPSC amplitudes, IPSC amplitudes, and their ratios following electrical LOT stimulation.

reflect the E/I balance. To measure this, we recorded synaptic currents in response to brief light pulses when cells were held at −70 mV to isolate EPSCs and at +5 mV to isolate disynaptic, feedback IPSCs (Figures 2F and 2G). Both responses were completely blocked by glutamate receptor antagonists (Figure S1G). Outward currents were also blocked by gabazine (GBZ), indicating that these were disynaptic IPSCs recruited by excitatory ChR2$^+$ inputs (data not shown, but see Figures 4B and 4C). The EPSC/IPSC ratio was markedly larger in OpK mice (cntl, 0.597 ± 0.0916, n = 13 cells/4 mice; OpK, 1.18 ± 0.113, n = 16 cells/7 mice; p = 4.34e−4, unpaired t test; Figure 2G), indicating that OpK did indeed disrupt the E/I balance. OpK did not alter intrinsic excitability in either ChR2$^+$ or ChR2$^-$ cells (Figure S3), consistent with Cela et al. (2019).

In some recordings, we also electrically activated mitral cell axons from the olfactory bulb using a bipolar stimulating electrode placed in the lateral olfactory tract (LOT). These inputs form excitatory synaptic connections onto the distal apical dendrites of PCx principal cells, as well as onto a distinct population of inhibitory interneurons located in layer I that provide feedforward inhibition onto principal cells (Figure 2E). This pathway is upstream of the optogenetically activated recurrent excitation/feedback inhibition pathway. The ratios of electrically evoked LOT EPSCs and disynaptic IPSCs from layer I feedforward interneurons were similar in cntl and OpK mice (cntl, 0.544 ± 0.0840, n = 13 cells/4 mice; OpK, 0.517 ± 0.0639, n = 9 cells/3 mice; p = 0.799, unpaired t test; Figure 2I), indicating that effects of OpK are pathway specific.

An increase in the E/I balance could be a consequence of an increase in the strength of recurrent excitation, a decrease in the strength of feedback inhibition, or both. Light-evoked EPSC amplitudes from cntl and OpK mice were similar (cntl, 316 ± 42.9 pA, n = 13 cells/4 mice; OpK, 308 ± 35.4 pA, n = 16 cells/7 mice; p = 0.879, unpaired t test; Figures 2F and 2G), whereas IPSCs were substantially smaller in slices from OpK versus cntl mice (cntl, 610 ± 76.1 pA; OpK, 295 ± 43.9 pA; p = 0.00189, unpaired t test), suggesting that OpK decreases synaptic inhibition. However, the comparison of evoked EPSC and IPSC amplitudes measured across cells from different animals is not a sufficiently robust quantification to convincingly localize the effect. We therefore examined the effects of optokindling on recurrent excitation and feedback inhibition more directly.

Optokindling does not strengthen recurrent excitation

OpK could increase the strength of recurrent excitation (1) by increasing presynaptic vesicle release probability, (2) by increasing the postsynaptic response to release of glutamate at existing synapses, or (3) by increasing the number of synaptic contacts between connected PCx neurons. We examined each of these possibilities separately. Paired-pulse ratios were equivalent across conditions (Figures 3A–3C), indicating that optokindling did not alter release probability. To examine postsynaptic response sensitivity, we measured quantal recurrent excitatory synaptic currents (qEPSCs) by using strontium substitution to desynchronize light-evoked vesicle release from ChR2$^+$ axons (Figure 3D). qEPSC amplitudes were equivalent across conditions (cntl, 20.3 ± 1.93 pA, n = 12 cells/4 mice; OpK, 24.1 ± 2.63, n = 12 cells/4 mice; p = 0.253, unpaired t test; Figures 3E–3G), indicating that OpK did not systematically strengthen recurrent synapses. Finally, we compared unitary excitatory synaptic currents (uEPSCs), which, given no change in qEPSC amplitude, could reflect changes in the number of synaptic connections between recurrently coupled neurons. To do this, we recorded synaptic responses in uninfected cells in response to prolonged, low-intensity light ramps that drove asynchronous spiking in ChR2$^+$ neurons and consequently produced uEPSCs in synaptically connected cells (Figure 3H). In control experiments, we verified that changing light intensity altered uEPSC frequency but did not affect uEPSC amplitude, consistent with these being unitary synaptic responses (Figure S4). However, again, we found no difference in uEPSC amplitudes (cntl, 21.1 ± 1.65 pA, n = 11 cells/5 mice; OpK, 24.1 ± 1.95 pA, n = 14 cells/4 mice; p = 0.242, unpaired t test; Figures 3I–3K), consistent with there being no increase in recurrent connectivity.

Optokindling depletes GABA and weakens feedback inhibition

The decreased amplitudes of evoked IPSCs suggest that feedback inhibition was weakened. To test this directly, we recorded miniature IPSCs (mIPSCs) in uninfected pyramidal cells (Figure 4A). We excluded mIPSCs with slower kinetics (full width at half-maximum > 8 ms) to select for perisomatic feedback inhibitory inputs and exclude inputs from feedforward inhibitory neurons in layer Ia that are driven by olfactory bulb inputs and primarily target apical dendrites (Stokes and Isaacson, 2010; Suzuki and Bekkers, 2010a). mIPSC amplitudes were smaller in OpK mice (cntl, 45.0 ± 2.85 pA, n = 14 cells/3 mice; OpK, 35.2 ± 3.66 pA, n = 21 cells/3 mice; p = 0.0205, unpaired t test; Figures 4B–4D). We also observed a decrease in mIPSC frequency in OpK mice (cntl, 6.90 ± 0.439 Hz; OpK, 4.08 ± 0.458 Hz; p = 9.90e−5, unpaired t test; Figure 4E). Taken together, our data indicate that the robust activation of uninfected PCx principal cells following OpK is not due to a systematic strengthening of excitatory connections from ChR2$^+$ onto ChR2$^-$ principal cells, but rather because of decreased feedback inhibition.

To determine whether the decreased mIPSC frequency simply reflected a loss of feedback inhibitory neurons following OpK, we performed an *in-situ* hybridization assay against the vesicular GABA transporter (*VGAT*; Figures 4F–4H). However, we observed similar numbers of layer I (i.e., feedforward) and layer III (i.e., feedback) interneurons in both aPCx and pPCx from OpK versus sham-injected cntl mice, indicating that OpK did not kill inhibitory interneurons. We did not count *VGAT*$^+$ neurons in layer II as axon terminal labeling made cell identification ambiguous.

We next asked whether OpK disrupted GABA expression. Mice were killed 24 h after either 1 or 6 days of optical stimulation, and we then stained for GABA and counted GABA$^+$ neurons (Figure 4I). In aPCx, at the site of optical stimulation, we observed a marked decrease in the number of GABA$^+$ neurons located in layers II and III in OpK mice (Figures 4I and 4J). The numbers of inhibitory neurons in layer I (i.e., feedforward interneurons, which are upstream of optogenetic activation) were equivalent

CellPress
OPEN ACCESS

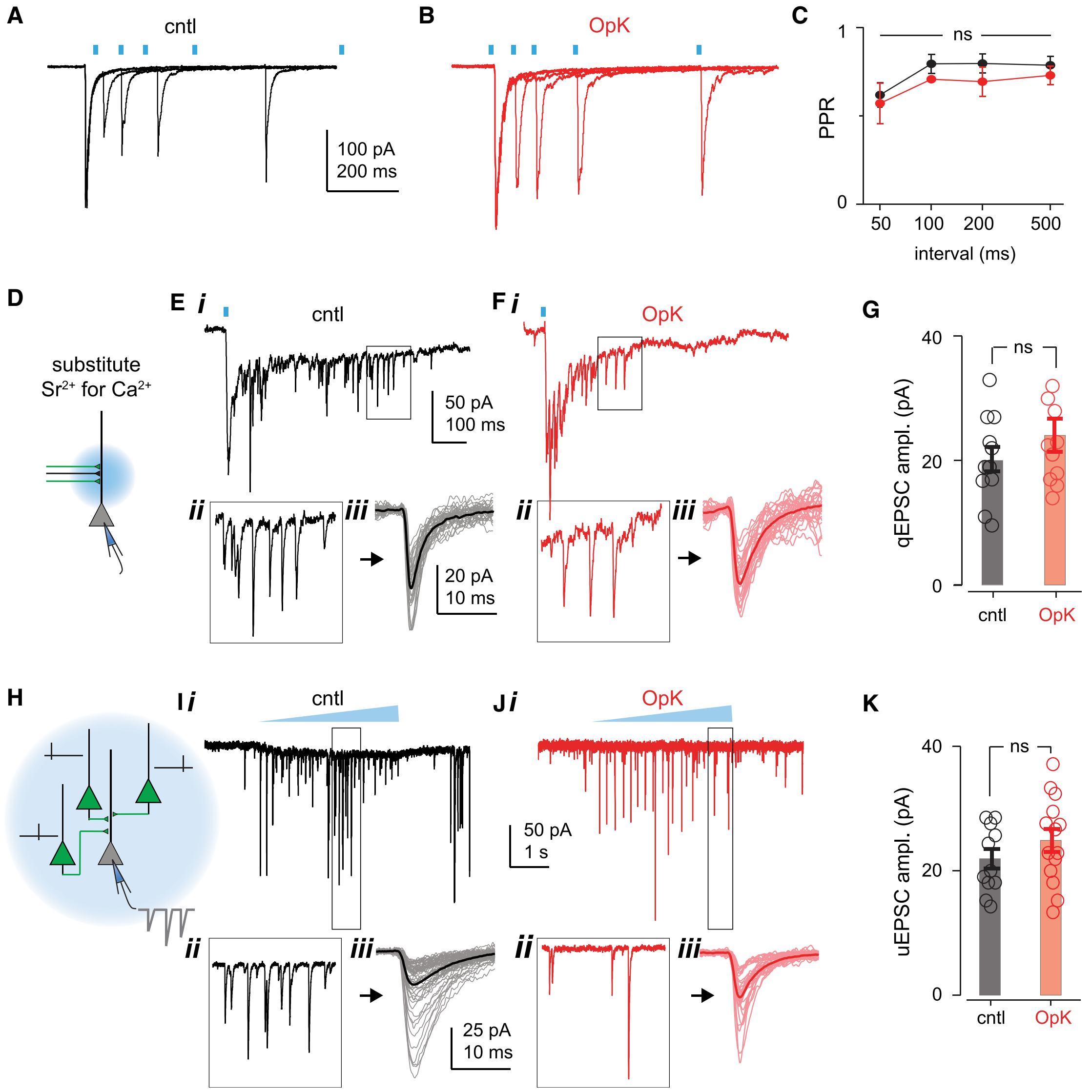

Figure 3. Optokindling does not strengthen recurrent excitation

(A–C) Equivalent paired-pulse ratios in cells from example of a cntl mouse (A) and an OpK mouse (B), and summary across mice (C; cntl, n = 8 cells from 3 mice; OpK, n = 8 cells from 3 mice; two-way ANOVA; $F(1,56) = 2.299$, $p = 0.136$) do not support changes in presynaptic release probability.

(D) Experimental schematic for evoking and measuring quantal EPSCs (qEPSCs) in uninfected neurons by 2-ms focal light pulses. Transmitter release is desynchronized when extracellular Ca^{2+} is replaced with Sr^{2+}.

(E) Example single Sr^{2+} trial trace in a cell from a cntl mouse (i) following optical activation (blue bar). Inset below (ii) shows the trace at an expanded scale corresponding to the boxed region in (i). Traces on right bottom (iii) show 30 sequentially recorded qEPSCs (thin traces) and the ensemble average response (thick traces) that was used to provide an average qEPSC amplitude for the example cell.

(F) As in (E) but for an example cell from an OpK mouse.

(G) Summary of all qEPSC amplitudes recorded from cntl and OpK mice. Mean qEPSC amplitudes for each cell (open circles) and average across experiments (bars).

(H) Experimental schematic for evoking and measuring unitary EPSCs (uEPSCs). Weak 5-s ramping light pulses evoke asynchronous spiking in $ChR2^{+}$ neurons (thin black ticks), which produce uEPSCs in postsynaptic $ChR2^{-}$ neurons (thicker gray trace).

(I) Example single trial in a cell from a cntl mouse (i) following weak, ramping light pulse activation (blue triangle). Inset below (ii) shows the trace at an expanded scale corresponding to the boxed region in (i). Traces on right bottom (iii) show 30 sequentially recorded uEPSCs (thin traces) and averaged response for all uEPSCs (thick traces) to provide an average uEPSC amplitude for the example cell.

(J) As in (I) but for an example cell from an OpK mouse.

(K) Summary of all uEPSCs recorded from cntl and OpK mice. Mean uEPSC amplitudes for each cell (open circles) and average across experiments (bars).

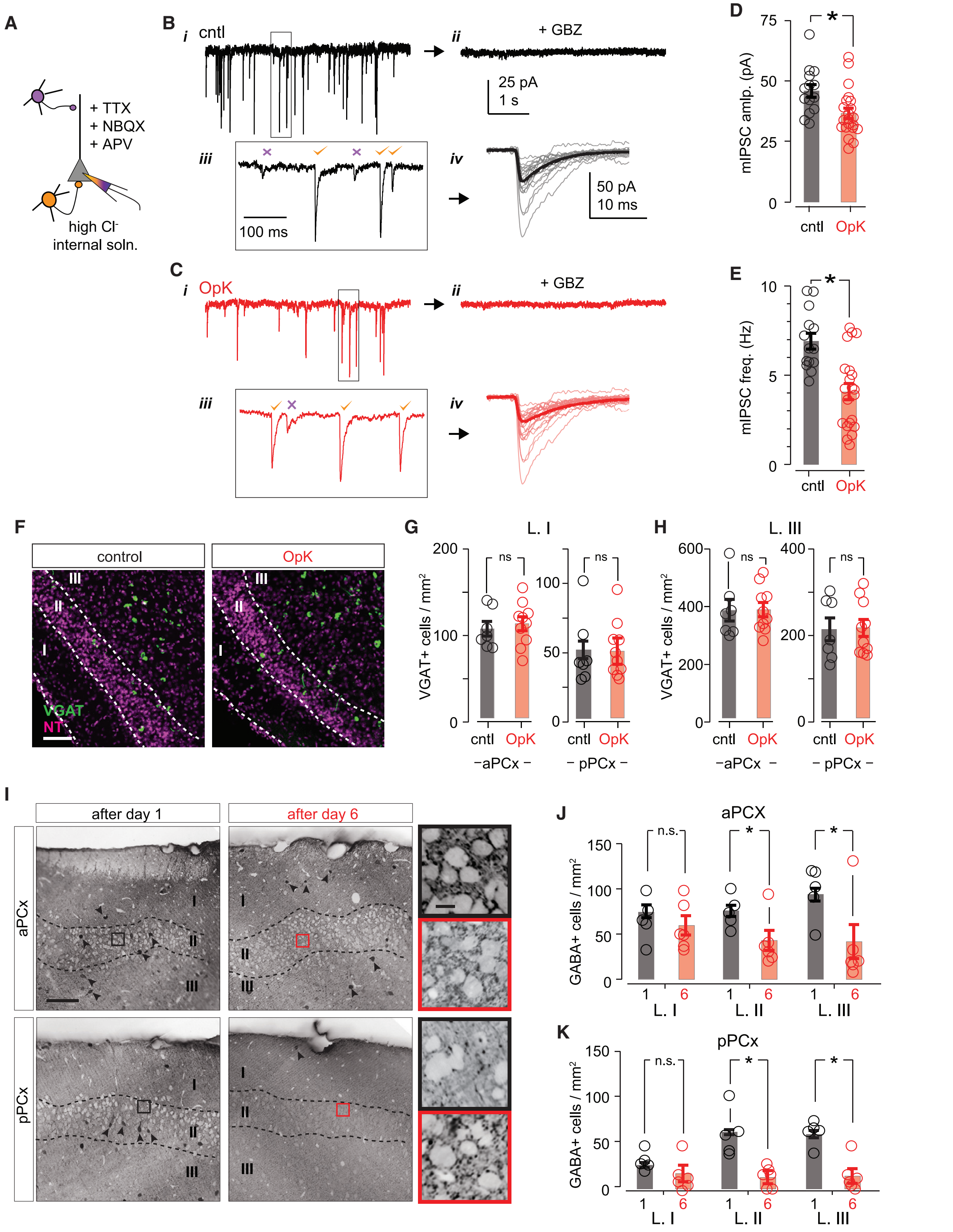

(legend on next page)

in cntl and OpK animals, consistent with electrophysiological results (Figures 2H and 2I). The loss of GABA labeling was even more pronounced in pPCx, downstream of the stimulation site (Figures 4I and 4K), where *Fos* labeling was most intense following seizures (Figures 2B and 2D). We observed clear and robust GABA staining in the hippocampus, neocortex, and lateral amygdala in the same slices, indicating that decreased GABA expression was restricted to PCx and was not a non-specific consequence of generalized seizures (Figure S5). Despite the depletion of somatic GABA staining, we nevertheless observed $GABA^+$ puncta ensheathing the somata of layer II principal neurons, where feedback inhibitory neurons contact their postsynaptic targets, in both cntl and OpK mice (Figure 4I, insets). Taken together, our data are consistent with a model in which OpK decreases the synthesis and/or availability of GABA. If so, we predict that although we see GABA-labeled puncta at presynaptic terminals, OpK should not affect GABA vesicle release mechanisms but may decrease synaptic GABA concentration and may affect the availability of GABA required for subsequent vesicle reloading.

To test whether OpK altered synaptic GABA concentrations, we directly activated feedback inhibitory neurons by electrically stimulating feedback inhibitory interneurons at the layer II/III boundary with excitatory synaptic transmission blocked (Figure 5A), and then measured the fractional block of the resultant IPSCs after the addition of 100 μM TPMPA ((1,2,5,6-Tetrahydropyridin-4-yl)methylphosphinic acid), a low-affinity competitive $GABA_A$ receptor antagonist (dissociation constant $[K_D]$ = 320 μM) (Jones et al., 2001) (Figure 5B). Synaptically released GABA displaces TPMPA from postsynaptic $GABA_A$ receptors, and so, if OpK decreases vesicle GABA concentrations, then IPSCs from OpK slices should be more sensitive to TPMPA than IPSCs from cntl slices (Clements et al., 1992; Jones et al., 2001). Indeed, residual IPSC amplitudes after adding TPMPA were markedly smaller in OpK versus cntl slices (cntl, 69.7% ± 4.32% of the baseline amplitude, n = 9 cells/3 mice; OpK, 46.8% ± 5.51%, n = 9 cells/3 mice; p = 0.0051, unpaired t test; Figure 5C). In both cases, the remaining currents were fully blocked by the addition of high concentrations (10 μM) of the high-affinity $GABA_A$ antagonist, SR-95531 (GBZ; Figure 5B). Importantly, the fractional IPSC block after addition of sub-saturating GBZ concentrations (100 nM) was equivalent in cntl and OpK slices (cntl, 45.3% ± 3.39%, n = 7 slices/4 mice; OpK, 37.9% ± 2.10%, n = 5 slices/2 mice; p = 0.098; Figures 5D and 5E).

In a separate set of experiments, we examined the dynamics of inhibitory synaptic transmission, again by electrically stimulating feedback inhibitory interneurons with excitatory synaptic transmission blocked (Figure 5F). We now presented 100 pulses at 20 Hz, similar to the *in vivo* optical stimulus protocol, although trains were presented every 30 s rather than every 10 min (Figure 5G). To determine whether OpK changes the release probability from layer II/III feedback interneurons onto layer II pyramidal cells, we examined the paired-pulse ratio by comparing the ratio of IPSC amplitudes for the second/first pulses (traces averaged across all 8 trials) but found no difference between cells from cntl and OpK mice (cntl, 0.716 ± 0.0263, n = 11 cells/3 mice; OpK, 0.738 ± 0.0390, n = 11 cells/3 mice; p = 0.638, unpaired t test, Figure 5H). Next, we asked whether OpK disrupted the size of the releasable vesicle pool by comparing the ratios of IPSC amplitudes for the 100th/1st pulses (traces averaged across all 8 trials) and again found no difference in OpK mice (cntl, 0.280 ± 0.0208; OpK, 0.351 ± 0.0327; p = 0.0823, unpaired t test; Figure 5I).

The amplitudes of the first IPSCs in each train were constant across trials in cntl mice but, interestingly, decreased with successive trials in OpK mice (mean ± SEM amplitudes of first pulses on eighth trial/first trial: cntl, 0.997 ± 0.109,

Figure 4. Optokindling depletes GABA and weakens feedback inhibition

(A) Experimental schematic for recording miniature IPSCs (mIPSCs). Fast IPSCs that originate from perisomatic-targeting feedback inhibitory interneurons (orange cells) were included; IPSCs from dendritic-targeting feedforward inhibitory interneurons (purple cells) were excluded based on their slow kinetics. Recordings were obtained using a high-Cl^- internal solution with sodium channels and glutamate receptors blocked.

(B) Example trace from a cntl mouse ($V_m = -70$ mV; i). Synaptic currents were completely blocked by gabazine (10 μM, ii). Insets on bottom show indicated region on top trace at an expanded timescale (iii). mIPSCs with fast (orange checks) and slow (purple crosses) kinetics were clearly distinguishable, and we excluded slow mIPCSs to select for feedback mIPSCs. (iv) Overlaid examples of the first 20 mIPSCs (thin traces) and average of all mIPSCs from that cell (thick trace).

(C) As in (B), but with an example from an OpK mouse.

(D) Summary of average mIPSC amplitudes measured from cntl and OpK mice. Open circles represent mean mIPSC amplitudes for each cell (cntl, 14 cells/3 mice; OpK, 21 cells/3 mice); bars indicate average mIPSC amplitudes across cells.

(E) As in (D), but for mIPSC frequency.

(F) Representative *VGAT in situ* hybridization images from pPCx of cntl (left) and OpK (right) mice. Dashed lines demarcate laminar boundaries, which are labeled by numerals. Scale bar: 100 μm.

(G) Number of L. I $VGAT^+$ neurons from cntl (black) and OpK (red) mice. Each point represents counts for one section, bars indicate average across sections, and error bars represent SEM. $VGAT^+$ cells in aPCx: cntl vs. OpK, p = 0.855; pPCx: cntl vs. OpK, p = 0.731. We used 7 sections from 2 mice for cntl and 10 sections from 3 mice for OpK analyses, and unpaired t tests were used in all cases.

(H) As in (G) but for L. III. aPCx: cntl vs. OpK, n = 830; lpPCx: cntl vs. OpK, p = 0.670.

(I) Representative sections of GABA-stained sections from mice stimulated for either 1 or 6 days; aPCx and pPCx sections are from the same mouse. Dashed lines demarcate different layers, which are indicated by numerals. Some GABA-stained neurons are indicated by arrowheads. Note the absence of GABA-stained neurons in layers II and III following OpK, especially in pPCx. Insets show magnified regions indicated by boxes, with contrast increased for visibility of perisomatic GABA puncta. Scale bars: 100 μm and 10 μm.

(J) Average numbers of $GABA^+$ neurons in each layer measured in aPCx after 1 (black) or 6 (red) days of stimulation. Circles represent the average cell counts for each animal across four sequential 50-μm sections. L. I: day 1 (n = 18 sections/5 mice) vs. day 6, (n = 23 sections/6 mice), p = 0.503, unpaired t test; L. II: day 1 vs. day 6 p = 0.00402; L. III: day 1 vs. day 6, p = 0.000209).

(K) As in (J), but for pPCx. L. I: day 1 vs. day 6, p = 0.106; L. II: day 1 vs. day 6, $p = 2.15 \times 10^{-5}$; L. III: day 1 vs. day 6, $p = 3.96 \times 10^{-5}$.

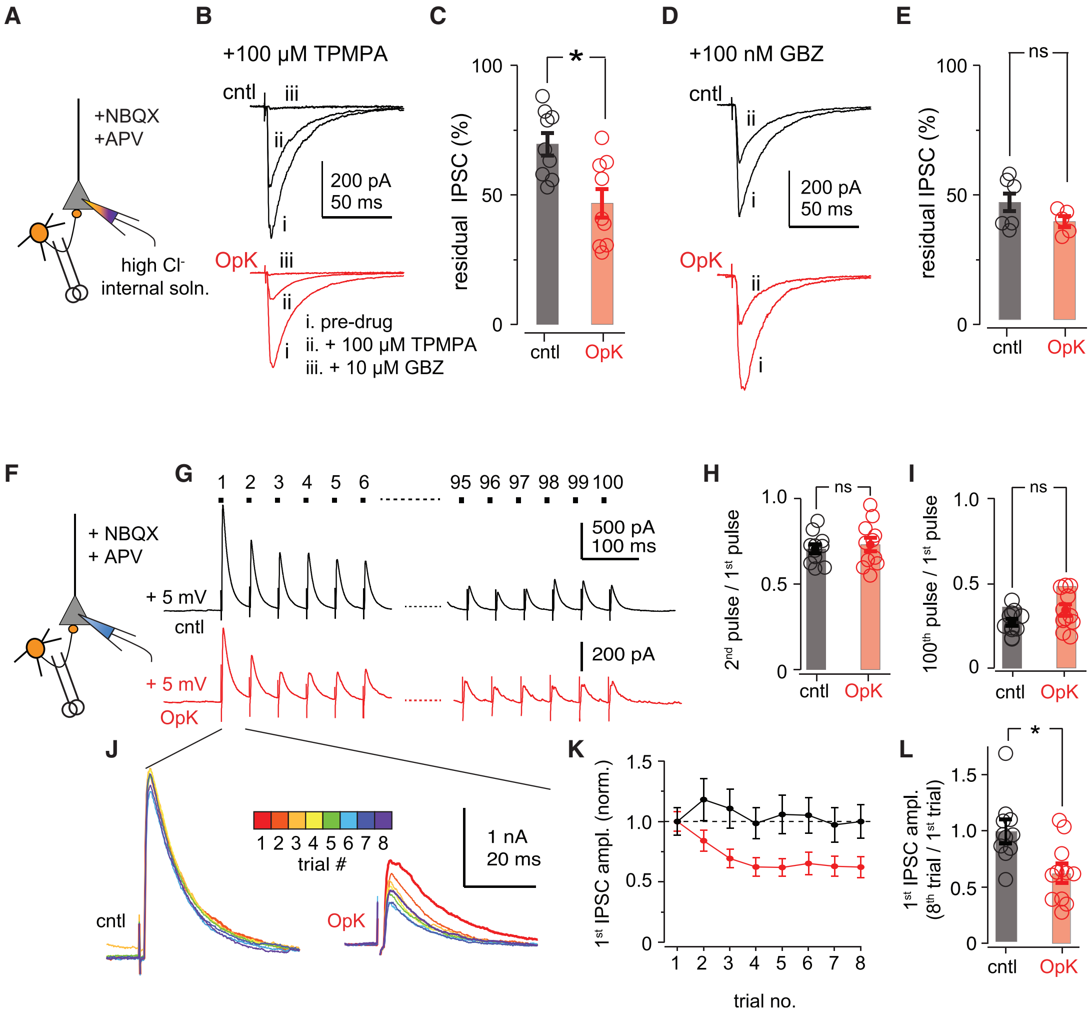

Figure 5. Optokindling decreases synaptic GABA concentration and slows vesicle refilling

(A) Experimental schematic. Direct IPSCs were evoked by electrically stimulating at the layer II/III boundary ~250 μm from the recorded cell with excitatory synaptic transmission blocked. Responses were recorded (V_m = −70 mV) with a high-chloride pipette solution, resulting in inward IPSCs.

(B) Example IPSCs from a cntl mouse (black traces on top) and an OpK mouse (red traces on bottom) in regular artificial cerebrospinal fluid (aCSF) (i), after addition of 100 μM TPMPA (ii), followed by addition of 10 μM GBZ (iii).

(C) Summary of residual IPSCs after addition of 100 μM TPMPA.

(D) Example IPSCs from a cntl mouse (black traces on top) and an OpK mouse (red traces on bottom) in regular aCSF (i), after addition of 100 nM GBZ (ii).

(E) Summary of residual percentage of IPSCs after addition of 100 nM GBZ.

(F) Schematic for experiments, as in (A), except responses were recorded (V_m = +5 mV) with a regular Cs-gluconate pipette solution, resulting in outward IPSCs.

(G) Example responses following 100 stimuli at 20 Hz in a cntl (top) and OpK (bottom) mouse. Traces represent averages of 8 sequential trials presented 30 s apart. Stimulus artifacts have been truncated for clarity. Note the different vertical scale bars of cntl and OpK traces.

(H) Paired-pulse ratios (50-ms interstimulus interval) calculated as the ratio of the second over the first IPSC amplitude.

(I) Measure of vesicle depletion calculated as the ratio of the 100th over the first IPSC amplitude.

(J) Overlays of the first IPSCs within a trial for each of the 8 trials that are averaged in (G). The amplitudes across trials are constant in cntl slices (left), whereas there is a progressive decrease in amplitude in slices from OpK mice (right).

(K) Average first IPSC ratios across trials, normalized to the amplitude on the first trial (cntl, 11 cells/3 mice; OpK, 12 cells/3 mice).

(L) Ratios of first IPSC amplitudes for the first and eighth trials.

n = 11 cells/3 mice, p = 0.994, paired t test; OpK, 0.624 ± 0.0742, n = 12 cells/3 mice, p = 0.00360; Figures 5J–5L). This result suggests that the 30-s interval between trials was no longer sufficient to accommodate the synthesis, refilling, and/or reloading of GABA into synaptic vesicles following OpK. Note again, however, that here we waited only 30 s between trains, as opposed to the 10-min interstimulus intervals used for *in vivo* stimulation. Nevertheless, these experiments indicate that OpK impaired the ability to replenish synaptic GABA following prolonged stimulation.

CellPress
OPEN ACCESS

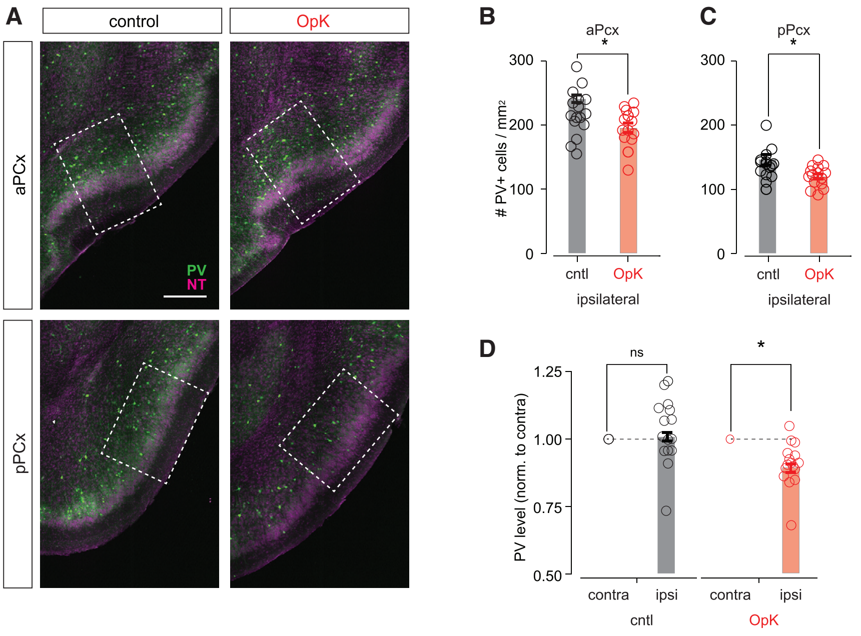

Figure 6. Optokindling decreases PV expression

(A) Representative sections showing PV expression (green) in aPCx (top) and pPCx (bottom) from a cntl mouse (left) and an OpK mouse (right). The dashed rectangles indicate the 500 μm × 700 μm areas that were used for counting PV$^+$ neurons. Scale bar, 300 μm. The NT counterstain is shown in magenta.

(B) Number of PV$^+$ neurons in aPCx (cntl, 241 ± 5.83 cells/mm^2, n = 16 slices/4 mice; OpK, 195 ± 7.38 cells/mm^2, n = 16 slices/4 mice; p = 0.043, unpaired t test).

(C) Number of PV$^+$ neurons in pPCx (cntl, 146 ± 8.34 cells/mm^2, n = 16 slices/4 mice; OpK, 120 ± 3.85 cells/mm^2, n = 16 slices/4 mice; p = 0.033, unpaired t test).

(D) Intensity of detected PV$^+$ somata in pPCx normalized to average intensity of identified PV$^+$ somata in the contralateral hemisphere (cntl, 1.05 ± 0.0309, n = 16 slices/4 mice; paired t test, 0.137; OpK, 0.905 ± 0.0209, n = 16 slices/4 mice; p = 0.00031).

Optokindling decreases parvalbumin expression

Layer III inhibitory interneurons in PCx are composed primarily of parvalbumin- (PV) and somatostatin (Sst)-expressing neurons (Suzuki and Bekkers, 2010b). We therefore attempted to use immunohistochemical markers to determine whether the effects of OpK are cell type specific or generalize across multiple classes. We were able to reliably stain for PV and observed slightly fewer PV$^+$ neurons in both the aPCx and pPCx of OpK mice (Figures 6A–6C); however, this effect, although statistically significant, was modest. Interestingly, we also noticed that PV$^+$ staining was less intense in the ipsilateral PCx of OpK mice, compared with PCx of the contralateral hemisphere (Figure 6D). This result suggests that the slight decrease in the number of PV$^+$ neurons does not reflect a loss, per se, of PV neurons, but rather that OpK downregulates PV expression levels in PCx, and that apparent differences in PV$^+$ cell counts may simply reflect thresholding error in counting. Intriguingly, our results may be a more extreme version of activity-dependent regulation of PV expression reported in the hippocampus (Donato et al., 2013, 2015). Unfortunately, we have not been able to label PCx neurons using various Sst or glutamate decarboxylase (GAD) antibodies (see STAR Methods; Figure S6).

Recurrent cortical circuits are especially prone to optokindling

Finally, we asked whether optokindling is specific to PCx. To do this, we repeated our experiments in four additional cohorts of Emx1-cre mice, with equivalent injections of Cre-dependent AAVs into either (1) PCx, (2) the CA3 region of the hippocampus, (3) primary somatosensory (S1), or (4) primary motor cortex (M1). We then repeated the optokindling procedures we used previously. Again, light intensity was titrated on the first day until animals only just appeared to notice the stimulus (i.e., usually by pausing briefly during stimulation). Once again previously innocuous optical stimulation of PCx principal neurons came to trigger pronounced seizures after just a few days of stimulation (Figure 7A). We obtained similar results in CA3, a similarly densely recurrent allocortical structure, indicating that OpK is not specific to PCx (Figure 7B), although kindling took slightly longer and seizures were slightly less robust than those in PCx (two-way ANOVA with replication, $F(1,22) = 6.29$, $p = 0.022$). However, we failed to evoke seizures in any of the mice following stimulation in either S1or M1 (Figures 7C and 7D). Note that Cela et al. (2019) were able to kindle mice by optogenetic stimulation of M1; however, their kindling paradigm required $\sim 10^2$ more stimuli before seizures were reliably observed. These data therefore indicate that highly recurrent circuits are especially prone to OpK.

DISCUSSION

The development of optogenetics has provided a powerful and precise tool for the study and potential treatment of epilepsy: a scalpel where before there was only a hammer (Krook-Magnuson and Soltesz, 2015). For the most part, these studies have generally used optogenetic tools for triggering (Khoshkhoo et al., 2017; Krook-Magnuson and Soltesz, 2015; Osawa et al., 2013; Wagner et al., 2015) or suppressing seizures (Krook-Magnuson et al., 2013, 2014; Paz et al., 2013; Sorokin et al., 2017; Sukhotinsky et al., 2013). Chemogenetic approaches are conceptually similar but with distinct advantages and disadvantages compared with optogenetic methods (Kätzel et al., 2014; Wicker and Forcelli, 2016). However, these studies have not typically used optogenetics to investigate underlying cellular mechanisms. Recently, an optogenetic model was developed showing that repeated *in vivo* activation of a targeted population of neocortical neurons can induce kindling in otherwise normal mice (Cela et al., 2019). We have extended this approach to

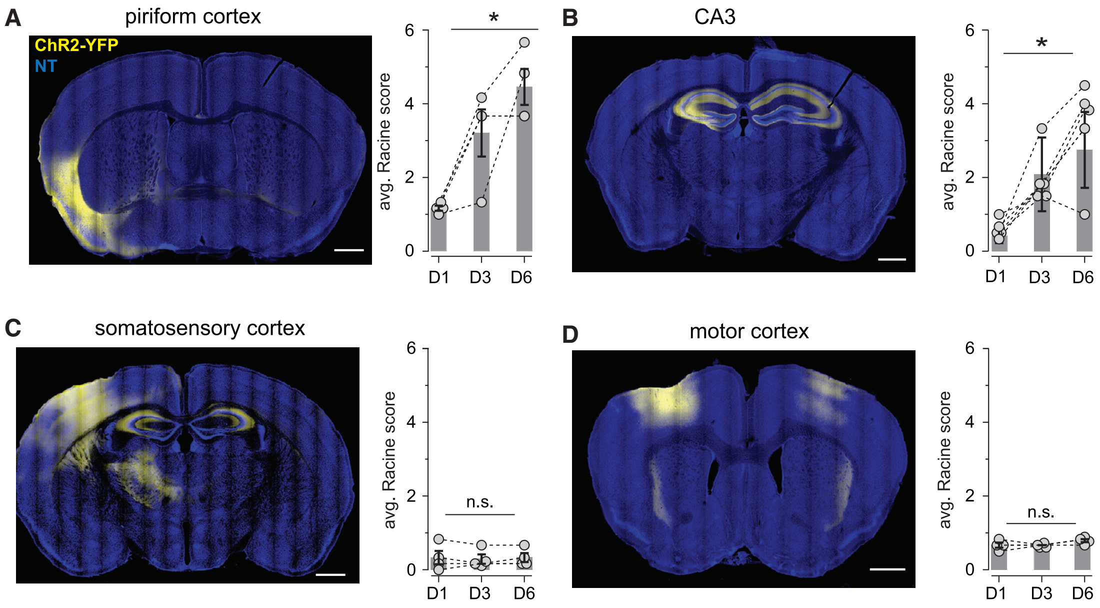

Figure 7. Recurrent circuits are especially prone to optokindling

(A, left) A representative coronal section showing ChR2-YFP expression in PCx. (right) Summary of change in Racine scores across days. Connected dots show the average scores across all six daily stimuli for each mouse, and bars show daily averages across mice (n = 4 mice; one-way ANOVA, $F(2,9) = 12.8$, $p = 0.00234$).

(B) As in (A) but for CA3 (n = 6; one-way ANOVA, $F(2,9) = 16.8$, $p = 0.000148$).

(C) As in (A) but for S1 (n = 4; one-way ANOVA, $F(2,9) = 0.0244$, $p = 0.975$).

(D) As in (A) but for M1 (n = 4; one-way ANOVA, $F(2,9) = 2.40$, $p = 0.147$).

All scale bars: 1 mm.

examine the cellular and synaptic processes that underlie kindling epileptogenesis.

Kindling in epilepsy research has been somewhat controversial. The major criticism of this model is that kindled mice do not generate spontaneous seizures, making this is a poor model for human epilepsy (Bertram, 2007). Although this criticism has some validity, spontaneous seizures eventually do emerge after extended kindling (Michalakis et al., 1998; Pinel, 1981). Nevertheless, an abundance of both clinical and experimental evidence indicates that seizures themselves are causal factors in progressive increases in severity. For example, catastrophic epileptic encephalopathies in children, including Dravet syndrome, which is usually caused by *de novo* mutations in *SCN1a* channels, are initially relatively benign but progressively increase in seizure severity, and this progression is thought to be caused by the seizures themselves. Similar progressive increases in seizure severity are often seen with temporal lobe epilepsy in adults. Kindling models are appropriate for understanding how this progressive epileptogenesis develops. A reliable and controlled way to repeatedly activate a targeted subset of neurons in an otherwise normal animal can therefore provide a powerful model for studying the role that seizures and seizure-like activity play in mediating their progressive increase in severity.

Therefore, we took advantage of the ability to selectively activate a defined population of cortical neurons *in vivo* and then re-activate the same neural populations *in vitro* to show that repeated activation of subsets of PCx principal neurons disrupts the balance of synaptic excitation and inhibition. This change in network excitability is pathway specific and occurs through a selective decrease in feedback inhibition. We find a decrease in the amplitude and frequency of mIPSCs, indicating a weakening of synaptic inhibition, consistent with some earlier findings following traditional kindling in the hippocampus (Kobayashi and Buckmaster, 2003; Wierenga and Wadman, 1999). However, our data are not consistent with other existing epileptogenesis models. First, we found equivalent numbers of *VGAT*$^{+}$ neurons in the *in-situ* hybridization assay, indicating that OpK does not result in pronounced cell death in inhibitory interneurons (Cossart et al., 2001; Dingledine et al., 2014; Dinocourt et al., 2003). Second, feedforward IPSCs, evoked by activating olfactory bulb inputs, in the same cells were unaffected. If OpK induced pronounced and cell-wide changes in intracellular chloride concentration in postsynaptic neurons (Cohen et al., 2002; Huberfeld et al., 2007; Kahle et al., 2008; Khalilov et al., 2003), then feedforward (layer I) and feedback (layers II and III) IPSCs would probably be affected equivalently (but see Chamma et al., 2012; Jedlicka et al., 2011; Waseem et al., 2010).

Our data suggest instead that inhibition is weakened by decreasing the expression, synthesis, and/or availability of GABA. First, both the amplitude and the frequency of mIPSCs were reduced after OpK, and synaptic GABA concentrations were decreased, as assessed by the fractional block of low-affinity competitive $GABA_A$ receptor antagonists. Second, somatic GABA expression was almost completely depleted in OpK mice 24 h after the last day of stimulation, although there were still $GABA^{+}$ puncta, surrounding cells in layer II, the main site of contact with principal cells (Stokes and Isaacson, 2010; Suzuki and

Bekkers, 2012). Third, we saw no changes in IPSC release probability (Figure 5H) or the size of the available pool of inhibitory synaptic vesicles (Figure 5I) but did see a progressive decrease in response amplitude on successive trains of stimuli, suggesting that vesicle refilling is impaired, which is predicted if GABA is in limited supply. Fourth, although we did not continuously monitor OpK mice, we never observed spontaneous seizures between bouts of optical stimulation in these mice, suggesting that inhibition is sufficiently robust to temper normal levels of excitation but cannot accommodate strong and prolonged excitation. Fifth, we rarely evoked seizures with the first stimulation train on any day, possibly reflecting sufficient time to reload GABA vesicles in the ~23 h since the last stimulus, but not at shorter intervals. Finally, shorter stimuli (1, 2, or 3 s long) did not evoke seizures, whereas 10-s-long stimuli triggered seizures almost every time, consistent with depletion of a limiting resource that checks runaway excitation. Note, however, that a slow depolarization block of fast-spiking interneurons could at least partially underlie this phenomenon (Cammarota et al., 2013). Taken together, these observations of GABA expression suggest that OpK impairs the synthesis of GABA at or near the soma, rather than its transport to axon terminals or loading into synaptic vesicles. Optokindling provides a targeted approach to identify the specific molecular-genetic mechanisms that underlie this phenomenon.

Our data are generally consistent with observations that reactive astrocytosis, which occurs with temporal lobe epilepsy, impairs GABA synthesis (Ortinski et al., 2010), and with evidence for decreased GABA labeling in the reserve vesicle pool, but not in the docked and readily releasable vesicle pools in perisomatic terminals of CA1 pyramidal cells of pilocarpine-treated rats (Hirsch et al., 1999). Interestingly, a previous study showed an activity-dependent, homeostatic balance of inhibitory synaptic transmission via bidirectional control of *Gad1* expression (Lau and Murthy, 2012). However, it remains unclear why and how processes that mediate homeostatic balance break down in epilepsy and epilepsy models (Staley, 2015).

Recurrent synaptic connections between PCx principal neurons undergo LTP and are thought to provide the substrate for odor learning and memory (Haberly, 2001; Wilson and Sullivan, 2011). Yet, somewhat surprisingly, we failed to detect any form of synaptic potentiation between $ChR2^+$ and $ChR2^-$ neurons (i.e., heterosynaptic LTP). However, our findings do not preclude selective potentiation between pairs of co-active $ChR2^+$ neurons (i.e., homosynaptic LTP), which are the connections that should be selectively strengthened according to Hebbian plasticity models (Dayan and Abbott, 2001). Examining changes in synaptic strength between $ChR2^+$ neurons is complicated by the fact that the synaptic currents recorded in postsynaptic cells will be occluded by the much larger photocurrents that are required to activate presynaptic axons. This obstacle can be overcome using the method we used here for measuring uEPSCs, in which stimulating light intensity is weak and the resulting photocurrents in $ChR2^+$ cells are relatively small (data not shown), providing a way to test the selective strengthening model in future studies.

We found that repeated optogenetic activation of ~10% of neurons in PCx results in kindling epileptogenesis that triggers massive, generalized seizures. Note that our expression levels are $>10^2$ greater than those of Choi et al. (2011), who paired optogenetic activation of ensembles of piriform neurons with aversive or appetitive conditioning and did not report seizures (Choi et al., 2011). However, odors also typically activate ~10% of PCx neurons (Bolding and Franks, 2017; Iurilli and Datta, 2017; Poo and Isaacson, 2009; Stettler and Axel, 2009) but rarely ever trigger seizures. Why are these scenarios so different? One possibility is that we optogenetically activated focal populations of infected cells, whereas odor-evoked ensembles are distributed across PCx (Illig and Haberly, 2003; Roland et al., 2017; Stettler and Axel, 2009). This distinction would be important if, as in neocortex, recurrent connectivity preferentially interconnected neighboring neurons. However, in recurrent allocortices, like PCx and CA3, neurons interconnect and synapse onto other principal cells and feedback interneurons with connection probabilities that are uniform over millimeter scales (Franks et al., 2011; Guzman et al., 2016; Johnson et al., 2000). Modeling studies show that focal and distributed activation patterns are effectively equivalent because of this long-range intrinsic connectivity (Guzman et al., 2016; Stern et al., 2018).

However, the temporal properties of odor-evoked and optogenetic activity patterns are dramatically different. Odor-activated PCx neurons typically fire brief bursts of action potentials with variable latencies that tile the 300- to 500-ms respiration cycle (Bolding and Franks, 2018; Miura et al., 2012). By contrast, optogenetic stimulation likely drives strong, synchronous, and sustained spiking in most or all $ChR2^+$ neurons. Our results now suggest that these artificially high levels of synchronous activity may not only be highly nonphysiological but can have grossly pathological effects in plastic neural circuits.

Interestingly, this difference in local recurrence in neocortex and global recurrence in allocortex may explain why we were readily able to optokindle PCx and CA3 in 6 days, with no kindling effect in the S1 or M1. Note that Cela et al. (2019) did optogenetically kindle the M1, but their paradigm took at least 15 sessions, each consisting of 6,750 pulses, which was much longer than the six sessions with a total of 600 pulses that we used. Thus, the long-range recurrent architecture of allocortical circuits allows distributed computations that cannot be performed with only local recurrence but may render the tissue much more susceptible to seizures, consistent with classical studies (Goddard, 1967; Löscher and Ebert, 1996). Extension of the OpK approach we describe here provides a means to identify the specific cellular and molecular processes that underlie this form of epileptogenesis.

STAR★METHODS

Detailed methods are provided in the online version of this paper and include the following:

- KEY RESOURCES TABLE
- RESOURCE AVAILABILITY
    - Lead contact
    - Materials availability
    - Data and code availability
- EXPERIMENTAL MODEL AND SUBJECT DETAILS

- METHOD DETAILS
  - Optical fiber and optrodes
  - Virus, viral injection, fiber/optrode implantation
  - *In vivo* photostimulation and kindling paradigm
  - Behavioral analysis of seizure severity
  - *In vivo* electrophysiology
  - *In vitro* electrophysiology
  - Immunohistochemistry
  - Fos, GABA and PV cell counting
  - Fluorescent *in situ* hybridization
- QUANTIFICATION AND STATISTICAL ANALYSIS

SUPPLEMENTAL INFORMATION

Supplemental information can be found online at https://doi.org/10.1016/j.celrep.2021.109001.

ACKNOWLEDGMENTS

We thank L. Glickfeld, C. Hull, and J. McNamara for helpful discussions, and A. Fleischmann, J. McNamara, C. Poo, and A. Schaefer for comments on the manuscript. H. McMullen, F. Abiodun, and S. Beriwell helped with histology and cell counting, and F. Wang generously provided resources and invaluable companionship. This work was supported by grants from the Edward Mallinckrodt, Jr. Foundation and the NIH (DC015525).

AUTHOR CONTRIBUTIONS

Conceptualization, K.M.F.; methodology, K.M.F.; validation, B.R., P.L., and K.M.F.; formal analysis, B.R., F.S.-V., P.L., E.R., M.L., and K.M.F.; investigation, B.R., S.N., F.S.-V., P.L., E.R., M.L., J.T., and K.M.F.; resources, J.T. and K.M.F.; data curation, B.R. and K.M.F.; writing – original draft preparation, K.M.F.; writing – review & editing, B.R., S.N., F.S.-V., and K.M.F.; visualization, B.R. and K.M.F.; supervision, project administration, and funding acquisition, K.M.F.

DECLARATION OF INTERESTS

The authors declare no competing interests.

Received: June 26, 2019
Revised: December 10, 2020
Accepted: March 24, 2021
Published: April 20, 2021

REFERENCES

Bertram, E. (2007). The relevance of kindling for human epilepsy. Epilepsia *48* (*Suppl 2*), 65–74.

Bolding, K.A., and Franks, K.M. (2017). Complementary codes for odor identity and intensity in olfactory cortex. eLife *6*, e22630.

Bolding, K.A., and Franks, K.M. (2018). Recurrent cortical circuits implement concentration-invariant odor coding. Science *361*, eaat6904.

Bolding, K.A., Nagappan, S., Han, B.X., Wang, F., and Franks, K.M. (2020). Recurrent circuitry is required to stabilize piriform cortex odor representations across brain states. eLife *9*, e53125.

Borg-Graham, L.J., Monier, C., and Frégnac, Y. (1998). Visual input evokes transient and strong shunting inhibition in visual cortical neurons. Nature *393*, 369–373.

Braitenberg, V., and Schüz, A. (1998). Cortex: Statistics and Geometry of Neuronal Connectivity (Springer-Verlag).

Cammarota, M., Losi, G., Chiavegato, A., Zonta, M., and Carmignoto, G. (2013). Fast spiking interneuron control of seizure propagation in a cortical slice model of focal epilepsy. J. Physiol. *591*, 807–822.

Cela, E., McFarlan, A.R., Chung, A.J., Wang, T., Chierzi, S., Murai, K.K., and Sjöström, P.J. (2019). An Optogenetic Kindling Model of Neocortical Epilepsy. Sci. Rep. *9*, 5236.

Chamma, I., Chevy, Q., Poncer, J.C., and Lévi, S. (2012). Role of the neuronal K-Cl co-transporter KCC2 in inhibitory and excitatory neurotransmission. Front. Cell. Neurosci. *6*, 5.

Choi, G.B., Stettler, D.D., Kallman, B.R., Bhaskar, S.T., Fleischmann, A., and Axel, R. (2011). Driving opposing behaviors with ensembles of piriform neurons. Cell *146*, 1004–1015.

Choy, J.M.C., Suzuki, N., Shima, Y., Budisantoso, T., Nelson, S.B., and Bekkers, J.M. (2017). Optogenetic Mapping of Intracortical Circuits Originating from Semilunar Cells in the Piriform Cortex. Cereb. Cortex *27*, 589–601.

Clements, J.D., Lester, R.A., Tong, G., Jahr, C.E., and Westbrook, G.L. (1992). The time course of glutamate in the synaptic cleft. Science *258*, 1498–1501.

Cohen, I., Navarro, V., Clemenceau, S., Baulac, M., and Miles, R. (2002). On the origin of interictal activity in human temporal lobe epilepsy in vitro. Science *298*, 1418–1421.

Cossart, R., Dinocourt, C., Hirsch, J.C., Merchan-Perez, A., De Felipe, J., Ben-Ari, Y., Esclapez, M., and Bernard, C. (2001). Dendritic but not somatic GABAergic inhibition is decreased in experimental epilepsy. Nat. Neurosci. *4*, 52–62.

Davison, I.G., and Ehlers, M.D. (2011). Neural circuit mechanisms for pattern detection and feature combination in olfactory cortex. Neuron *70*, 82–94.

Dayan, P., and Abbott, L. (2001). Theoretical neuroscience computational and mathematical modeling of neural systems. Computational Neuroscience (MIT Press), pp. 281–328.

Dingledine, R., Varvel, N.H., and Dudek, F.E. (2014). When and how do seizures kill neurons, and is cell death relevant to epileptogenesis? Adv. Exp. Med. Biol. *813*, 109–122.

Dinocourt, C., Petanjek, Z., Freund, T.F., Ben-Ari, Y., and Esclapez, M. (2003). Loss of interneurons innervating pyramidal cell dendrites and axon initial segments in the CA1 region of the hippocampus following pilocarpine-induced seizures. J. Comp. Neurol. *459*, 407–425.

Donato, F., Rompani, S.B., and Caroni, P. (2013). Parvalbumin-expressing basket-cell network plasticity induced by experience regulates adult learning. Nature *504*, 272–276.

Donato, F., Chowdhury, A., Lahr, M., and Caroni, P. (2015). Early- and late-born parvalbumin basket cell subpopulations exhibiting distinct regulation and roles in learning. Neuron *85*, 770–786.

Douglas, R.J., Koch, C., Mahowald, M., Martin, K.A., and Suarez, H.H. (1995). Recurrent excitation in neocortical circuits. Science *269*, 981–985.

Franks, K.M., Russo, M.J., Sosulski, D.L., Mulligan, A.A., Siegelbaum, S.A., and Axel, R. (2011). Recurrent circuitry dynamically shapes the activation of piriform cortex. Neuron *72*, 49–56.

Goddard, G.V. (1967). Development of epileptic seizures through brain stimulation at low intensity. Nature *214*, 1020–1021.

Goddard, G.V., and Douglas, R.M. (1975). Does the engram of kindling model the engram of normal long term memory? Can. J. Neurol. Sci. *2*, 385–394.

Gowers, W.R. (1881). Epilepsy and other convulsive diseases (J & A Churchill).

Guzman, S.J., Schlögl, A., Frotscher, M., and Jonas, P. (2016). Synaptic mechanisms of pattern completion in the hippocampal CA3 network. Science *353*, 1117–1123.

Haberly, L.B. (2001). Parallel-distributed processing in olfactory cortex: new insights from morphological and physiological analysis of neuronal circuitry. Chem. Senses *26*, 551–576.

Hagiwara, A., Pal, S.K., Sato, T.F., Wienisch, M., and Murthy, V.N. (2012). Optophysiological analysis of associational circuits in the olfactory cortex. Front. Neural Circuits *6*, 18.

Haider, B., Duque, A., Hasenstaub, A.R., and McCormick, D.A. (2006). Neocortical network activity in vivo is generated through a dynamic balance of excitation and inhibition. J. Neurosci. *26*, 4535–4545.

CellPress
OPEN ACCESS

Hirsch, J.C., Agassandian, C., Merchán-Pérez, A., Ben-Ari, Y., DeFelipe, J., Esclapez, M., and Bernard, C. (1999). Deficit of quantal release of GABA in experimental models of temporal lobe epilepsy. Nat. Neurosci. *2*, 499–500.

Huberfeld, G., Wittner, L., Clemenceau, S., Baulac, M., Kaila, K., Miles, R., and Rivera, C. (2007). Perturbed chloride homeostasis and GABAergic signaling in human temporal lobe epilepsy. J. Neurosci. *27*, 9866–9873.

Illig, K.R., and Haberly, L.B. (2003). Odor-evoked activity is spatially distributed in piriform cortex. J. Comp. Neurol. *457*, 361–373.

Iurilli, G., and Datta, S.R. (2017). Population Coding in an Innately Relevant Olfactory Area. Neuron *93*, 1180–1197.e7.

Jedlicka, P., Deller, T., Gutkin, B.S., and Backus, K.H. (2011). Activity-dependent intracellular chloride accumulation and diffusion controls GABA(A) receptor-mediated synaptic transmission. Hippocampus *21*, 885–898.

Johnson, D.M.G., Illig, K.R., Behan, M., and Haberly, L.B. (2000). New features of connectivity in piriform cortex visualized by intracellular injection of pyramidal cells suggest that "primary" olfactory cortex functions like "association" cortex in other sensory systems. J. Neurosci. *20*, 6974–6982.

Jones, M.V., Jonas, P., Sahara, Y., and Westbrook, G.L. (2001). Microscopic kinetics and energetics distinguish GABA(A) receptor agonists from antagonists. Biophys. J. *81*, 2660–2670.

Jung, M.W., Larson, J., and Lynch, G. (1990). Long-term potentiation of monosynaptic EPSPs in rat piriform cortex in vitro. Synapse *6*, 279–283.

Kahle, K.T., Staley, K.J., Nahed, B.V., Gamba, G., Hebert, S.C., Lifton, R.P., and Mount, D.B. (2008). Roles of the cation-chloride cotransporters in neurological disease. Nat. Clin. Pract. Neurol. *4*, 490–503.

Kanter, E.D., and Haberly, L.B. (1990). NMDA-dependent induction of long-term potentiation in afferent and association fiber systems of piriform cortex in vitro. Brain Res. *525*, 175–179.

Kätzel, D., Nicholson, E., Schorge, S., Walker, M.C., and Kullmann, D.M. (2014). Chemical-genetic attenuation of focal neocortical seizures. Nat. Commun. *5*, 3847.

Khalilov, I., Holmes, G.L., and Ben-Ari, Y. (2003). In vitro formation of a secondary epileptogenic mirror focus by interhippocampal propagation of seizures. Nat. Neurosci. *6*, 1079–1085.

Khoshkhoo, S., Vogt, D., and Sohal, V.S. (2017). Dynamic, Cell-Type-Specific Roles for GABAergic Interneurons in a Mouse Model of Optogenetically Inducible Seizures. Neuron *93*, 291–298.

Kobayashi, M., and Buckmaster, P.S. (2003). Reduced inhibition of dentate granule cells in a model of temporal lobe epilepsy. J. Neurosci. *23*, 2440–2452.

Krook-Magnuson, E., and Soltesz, I. (2015). Beyond the hammer and the scalpel: selective circuit control for the epilepsies. Nat. Neurosci. *18*, 331–338.

Krook-Magnuson, E., Armstrong, C., Oijala, M., and Soltesz, I. (2013). On-demand optogenetic control of spontaneous seizures in temporal lobe epilepsy. Nat. Commun. *4*, 1376.

Krook-Magnuson, E., Szabo, G.G., Armstrong, C., Oijala, M., and Soltesz, I. (2014). Cerebellar Directed Optogenetic Intervention Inhibits Spontaneous Hippocampal Seizures in a Mouse Model of Temporal Lobe Epilepsy. eNeuro *1*, ENEURO.0005-14.2014.

Large, A.M., Vogler, N.W., Mielo, S., and Oswald, A.M. (2016). Balanced feedforward inhibition and dominant recurrent inhibition in olfactory cortex. Proc. Natl. Acad. Sci. USA *113*, 2276–2281.

Lau, C.G., and Murthy, V.N. (2012). Activity-dependent regulation of inhibition via GAD67. J. Neurosci. *32*, 8521–8531.

Löscher, W., and Ebert, U. (1996). The role of the piriform cortex in kindling. Prog. Neurobiol. *50*, 427–481.

McNamara, J.O. (1995). Analyses of the molecular basis of kindling development. Psychiatry Clin. Neurosci. *49*, S175–S178.

Michalakis, M., Holsinger, D., Ikeda-Douglas, C., Cammisuli, S., Ferbinteanu, J., DeSouza, C., DeSouza, S., Fecteau, J., Racine, R.J., and Milgram, N.W. (1998). Development of spontaneous seizures over extended electrical kindling. I. Electrographic, behavioral, and transfer kindling correlates. Brain Res. *793*, 197–211.

Miura, K., Mainen, Z.F., and Uchida, N. (2012). Odor representations in olfactory cortex: distributed rate coding and decorrelated population activity. Neuron *74*, 1087–1098.

Ortinski, P.I., Dong, J., Mungenast, A., Yue, C., Takano, H., Watson, D.J., Haydon, P.G., and Coulter, D.A. (2010). Selective induction of astrocytic gliosis generates deficits in neuronal inhibition. Nat. Neurosci. *13*, 584–591.

Osawa, S., Iwasaki, M., Hosaka, R., Matsuzaka, Y., Tomita, H., Ishizuka, T., Sugano, E., Okumura, E., Yawo, H., Nakasato, N., et al. (2013). Optogenetically induced seizure and the longitudinal hippocampal network dynamics. PLoS ONE *8*, e60928.

Pashkovski, S.L., Iurilli, G., Brann, D., Chicharro, D., Drummey, K., Franks, K.M., Panzeri, S., and Datta, S.R. (2020). Structure and flexibility in cortical representations of odour space. Nature *583*, 253–258.

Paz, J.T., Davidson, T.J., Frechette, E.S., Delord, B., Parada, I., Peng, K., Deisseroth, K., and Huguenard, J.R. (2013). Closed-loop optogenetic control of thalamus as a tool for interrupting seizures after cortical injury. Nat. Neurosci. *16*, 64–70.

Pinel, J.P. (1981). Kindling-induced experimental epilepsy in rats: cortical stimulation. Exp. Neurol. *72*, 559–569.

Poo, C., and Isaacson, J.S. (2007). An early critical period for long-term plasticity and structural modification of sensory synapses in olfactory cortex. J. Neurosci. *27*, 7553–7558.

Poo, C., and Isaacson, J.S. (2009). Odor representations in olfactory cortex: "sparse" coding, global inhibition, and oscillations. Neuron *62*, 850–861.

Poo, C., and Isaacson, J.S. (2011). A major role for intracortical circuits in the strength and tuning of odor-evoked excitation in olfactory cortex. Neuron *72*, 41–48.

Rennaker, R.L., Chen, C.F., Ruyle, A.M., Sloan, A.M., and Wilson, D.A. (2007). Spatial and temporal distribution of odorant-evoked activity in the piriform cortex. J. Neurosci. *27*, 1534–1542.

Roland, B., Deneux, T., Franks, K.M., Bathellier, B., and Fleischmann, A. (2017). Odor identity coding by distributed ensembles of neurons in the mouse olfactory cortex. eLife *6*, e26337.

Shadlen, M.N., and Newsome, W.T. (1998). The variable discharge of cortical neurons: implications for connectivity, computation, and information coding. J. Neurosci. *18*, 3870–3896.

Shu, Y., Hasenstaub, A., and McCormick, D.A. (2003). Turning on and off recurrent balanced cortical activity. Nature *423*, 288–293.

Sorokin, J.M., Davidson, T.J., Frechette, E., Abramian, A.M., Deisseroth, K., Huguenard, J.R., and Paz, J.T. (2017). Bidirectional Control of Generalized Epilepsy Networks via Rapid Real-Time Switching of Firing Mode. Neuron *93*, 194–210.

Srinivasan, S., and Stevens, C.F. (2018). The distributed circuit within the piriform cortex makes odor discrimination robust. J. Comp. Neurol. *526*, 2725–2743.

Staley, K. (2015). Molecular mechanisms of epilepsy. Nat. Neurosci. *18*, 367–372.

Stern, M., Bolding, K.A., Abbott, L.F., and Franks, K.M. (2018). A transformation from temporal to ensemble coding in a model of piriform cortex. eLife *7*, e34831.

Stettler, D.D., and Axel, R. (2009). Representations of odor in the piriform cortex. Neuron *63*, 854–864.

Stokes, C.C.A., and Isaacson, J.S. (2010). From dendrite to soma: dynamic routing of inhibition by complementary interneuron microcircuits in olfactory cortex. Neuron *67*, 452–465.

Sukhotinsky, I., Chan, A.M., Ahmed, O.J., Rao, V.R., Gradinaru, V., Ramakrishnan, C., Deisseroth, K., Majewska, A.K., and Cash, S.S. (2013). Optogenetic delay of status epilepticus onset in an in vivo rodent epilepsy model. PLoS ONE *8*, e62013.

Suzuki, N., and Bekkers, J.M. (2010a). Distinctive classes of GABAergic interneurons provide layer-specific phasic inhibition in the anterior piriform cortex. Cereb. Cortex *20*, 2971–2984.

Suzuki, N., and Bekkers, J.M. (2010b). Inhibitory neurons in the anterior piriform cortex of the mouse: classification using molecular markers. J. Comp. Neurol. *518*, 1670–1687.

Suzuki, N., and Bekkers, J.M. (2012). Microcircuits mediating feedforward and feedback synaptic inhibition in the piriform cortex. J. Neurosci. *32*, 919–931.

van Vreeswijk, C., and Sompolinsky, H. (1996). Chaos in neuronal networks with balanced excitatory and inhibitory activity. Science *274*, 1724–1726.

Vaughan, D.N., and Jackson, G.D. (2014). The piriform cortex and human focal epilepsy. Front. Neurol. *5*, 259.

Wagner, F.B., Truccolo, W., Wang, J., and Nurmikko, A.V. (2015). Spatiotemporal dynamics of optogenetically induced and spontaneous seizure transitions in primary generalized epilepsy. J. Neurophysiol. *113*, 2321–2341.

Waseem, T., Mukhtarov, M., Buldakova, S., Medina, I., and Bregestovski, P. (2010). Genetically encoded Cl-Sensor as a tool for monitoring of Cl-dependent processes in small neuronal compartments. J. Neurosci. Methods *193*, 14–23.

Wicker, E., and Forcelli, P.A. (2016). Chemogenetic silencing of the midline and intralaminar thalamus blocks amygdala-kindled seizures. Exp. Neurol. *283* (*Pt A*), 404–412.

Wierenga, C.J., and Wadman, W.J. (1999). Miniature inhibitory postsynaptic currents in CA1 pyramidal neurons after kindling epileptogenesis. J. Neurophysiol. *82*, 1352–1362.

Wilson, D.A., and Sullivan, R.M. (2011). Cortical processing of odor objects. Neuron *72*, 506–519.

STAR★METHODS

KEY RESOURCES TABLE

REAGENT or RESOURCE	SOURCE	IDENTIFIER
Bacterial and virus strains		
AAV2/5-EF1a-DIO-hChR2(H134R)-EYFP	University of North Carolina Vector Core	N/A
AAV-EF1a-DIO hChR2 (E123T/T159C)-p2A-EYFP-WPRE	University of North Carolina Vector Core	N/A
Chemicals, peptides, and recombinant proteins		
Chicken anti-GFP Antibody	Abcam	RRID:AB_300798
Rabbit anti-Fos Antibody	Cell Signaling Technology	RRID:AB_2247211
Rabbit GABA Polyclonal Antibody	Invitrogen	RRID:AB_2549714
Rabbit PV Antibody	Invitrogen	RRID:AB_2173898
Rabbit SSt Antibody	Invitrogen	RRID:AB_2633039
Rabbit GAD Antibody	Cell Signaling Technology	RRID:AB_10835855
Goat Alexa 488 anti-Chicken Antibody	Abcam	RRID:AB_2636803
Alexa-555 anti-Rabbit Antibody	Invitrogen	RRID:AB_2536100
NeuroTrace 640	Invitrogen	RRID:AB_2572212
Sheep Anti-Digoxigenin Antibody	Millipore Sigma	RRID:AB_2734716
Anti-GFP Antibody	Abcam	RRID:AB_303395
Fast Red TR/Naphthol AS/MX	Sigma-Aldrich	F4648
Donkey Alexa 488 anti-Rabbit Secondary Antibody	Jackson ImmunoResearch Laboratories	RRID:AB_2313584
Vectashield	Vector Laboratories	N/A
Fluoromount-G	Thermo-Fisher	00-4958-02
TTX	Tocris	1078
NBQX	Tocris	0373
D-APV	Tocris	0106
Gabazine	Tocris	1262
TPMPA	Tocris	1040
Experimental models: Organisms/strains		
Emx1-Cre Mice	The Jackson Laboratory	RRID:IMSR_JAX:005628
Software and algorithms		
Spike2	Cambridge Electronic Design	N/A
MATLAB	MathWorks	N/A
Igor Pro	WaveMetrics	N/A
AxoGraphX	AxoGraph	N/A
ImageJ	Fiji	N/A
Other		
Optical Fiber Patch Cable	Thorlabs	MR81L01
Optical Fiber for Optrode	Thorlabs	FT200EMT
Ceramic Ferrule for Optrode	Precision Fiber Products	MM-FER2002S15
Silver Wire for Optrode	A-M Systems	786500
Connector Pins for Optrode	A-M Systems	520200
473 nm Laser	Shanghai Laser & Optics Century	N/A
Laser Power Meter	Thorlabs	PM100D
Laser Power Sensor	Thorlabs	S121C
Metabond	Parkell	S398, S371 and S399
AC/DC Differential Amplifier	A-M Systems	Model 3000
Data Acquisition System	Cambridge Electronic Design	Power1401-3A

RESOURCE AVAILABILITY

Lead contact

Further information and requests for resources and reagents should be directed to and will be fulfilled by the lead contact, Kevin Franks (franks@neuro.duke.edu).

Materials availability

This study did not generate new unique reagents.

Data and code availability

This study did not generate/analyze any datasets/code. Reasonable requests for raw data from the study should be directed to and will be fulfilled by the lead contact, Kevin Franks (franks@neuro.duke.edu)

EXPERIMENTAL MODEL AND SUBJECT DETAILS

Juvenile and young adult mice (male and female, 3-12 weeks) were used in this study and no attempt was made to determine if the sex of the animal had any impact on the results presented in the study. Animals were handled according to the National Institutes of Health Guide for the Care and Use of the Laboratory Animals and the experiments were conducted under an approved protocol by the Duke University Animal Care and Use Committee. All animals used in this study derived from breeding pairs of Emx1-Cre mice (JAX 005628). Mice were housed on a 12-hour light/dark cycle and had *ad libitum* access to food and water.

METHOD DETAILS

Optical fiber and optrodes

Optical fibers and optrodes were constructed as described in [79]. Briefly, optical fibers (0.39 NA, 200 μm diameter OM, Thorlabs) were threaded through a 2.5 mm ceramic ferrule (Precision Fiber Products, Inc.) and secured with epoxy. The end of the optical fiber at the ferrule base was polished with fiber polishing films (Thorlabs) and the other end was cut to 5 mm with a ruby fiber scribe (Thorlabs). Optrodes were optical fibers coupled with bipolar electrodes for LFP recording. These were constructed by bonding a twisted pair of insulated silver wires to the optical fiber protruding from the ferrule (wire: 0.011" diameter, #786500, AM Systems; connector pins: # 520200, AM Systems). Fibers were connected to a 473 nm laser (Shanghai Laser & Optic Century) via a standard fiber optical cable (Thorabs). Transmission efficiency through each optical fiber was measured prior to implantation. Each day, prior to stimulation, laser power was measured at the end of the optical patch cable (Thorlabs PM100D power meter with a S121C sensor), and then scaled by the transmission efficiency through the ferrule.

Virus, viral injection, fiber/optrode implantation

For selective channelrhodopsin-2 (ChR2) expression in PCx principal neurons, we injected AAV2/5-EF1a-DIO-hChR2(H134R)-EYFP or AAV-EF1a-DIO hChR2 (E123T/T159C)-p2A-EYFP-WPRE (both $\sim 10^{13}$ GC/mL; UNC Vector Core) into aPCx of Emx1-Cre mice (AP: −0.5 mm; ML: 3.5-3.6 mm; DV: 4.0 mm). Mice were lightly sedated using ketamine/xylazine (10 mg/kg:1 mg/kg) and kept under anesthesia with isoflurane after being placed in a stereotaxic frame. A small craniotomy was performed above one anterior piriform cortex. 250-300 nL of AAVs were introduced through a glass pipette using a nanoject (Drummond) at approximately 60 nl/min. The glass pipette was left in place for approximately 10 minutes after virus injection and then withdrawn slowly. Afterward, the optical fiber or optrode was slowly lowered to a depth approximately 300 μm above the injection site and cemented in place using metabond. For experimental mice with optrodes, a small piece of insulated silver wire connected to a gold pin reference electrode was placed in the skull over the cerebellum. Optic fiber/optrode implant location and viral expression were histologically verified after the behavioral studies. Only the mice with correct locations of implants and viral expression were analyzed. The following stereotaxic coordinates were used to other areas, with surgical procedures as described above and the kindling paradigm as described below: CA3 (AP: −1.6 mm; ML: 2.4 mm; DV: 2.1 mm); M1 (1.5 mm, 1.8 mm, −0.8 mm); S1 (−1.0 mm, 2.8 mm, −0.8 mm).

In vivo photostimulation and kindling paradigm

In vivo photostimulation began at least three weeks after injection to allow for robust and stable virus expression. Each animal was placed in an empty cage and habituated for ~10 minutes before stimulation, and then stimulatned for six consecutive days (6 times per day, each 10 minutes apart). To stimulate, optical fibers were connected through a ceramic split sleeve via FC/PC cables and a commutator (Thorlabs) to the laser. Initial laser power delivered to the tissue was 1 mW, after correcting for transmission loss through the optical fiber. Power was then titrated (0.5-2 mW) on the first day of stimulation until the animal paused briefly during light stimulation; this level of stimulation typically had little other effect on either the LFP or behavior and was almost always subconvulsive. Video was captured with a CCD camera (Firefly MV, Point Grey) synced to LFP recordings using the Spike2 Video Recorder tool. Photostimulation was controlled by Spike2 and consisted of a light train with 10 ms pulses delivered at 20 Hz for 5 s (i.e., 100 pulses).

After the initial bach of 23 mice, some animals occasionally missed one day of stimulation but this had no effect on kindling rate, and kindling rate is therefore described here in terms of days of stimulation rather than days since first stimulated.

Behavioral analysis of seizure severity

Kindling development was monitored and recorded in all animals by behavioral phenotype using a modified Racine scale. Seizure levels were classified as follows:

0, brief pause during light train
1, freezing, mild facial twitching
2, head nodding, oral twitching
3, single forelimb clonus
4, clonus in both forelimbs, rearing
5, rearing and falling on side
6, running jumping seizures.

Mice were considered fully kindled when stimulation evoked class 4 or greater seizures on at least three of the six daily stimuli. In initial experiments in which we quantified the evolution of optokindling (i.e., Figure 1, primarily), seizures were scored in real time by a cohort of 3 trained undergraduate observers, and videos were viewed and confirmed post hoc by BR and/or PL to ensure scoring was consistent across observers. Observers were not blinded to stimulation day. For later experiments, after the evolution of the OpK was well described, kindling was induced and scored by BR or FSV and we simply ensured each animal was kindled (i.e., $\geq$ 4 seizures on at least 3 of 6 stimuli on any one day).

In vivo electrophysiology

Local field potentials were measured in animals with optrode implants by connecting the optrode, through an electrical commutator (PlasticsOne), to an amplifier (Model 3000, A&M Systems; amplified 100x), filtered (high-pass, 1 Hz; low-pass 300 Hz), and digitized at 3 kHz; (Power 1401, Cambridge Electronic Design) and recorded using Spike2 (CED). LFP data was recorded starting 10 s prior to, and one minute after photostimulation. Spike2 and IgorPro were used to analyze and visualize the data.

In vitro electrophysiology

All slice physiology experiments were performed at least one month after virus injection (kindled: 42 ± 2.0 days after injection, range 33-51: cntl: 37 ± 2.4 days, range 30-44 days). Mice were anesthetized with isoflurane and decapitated, and the cortex was quickly removed in ice-cold artificial CSF (aCSF). Parasagittal brain slices (300 μm) were cut using a vibrating microtome (Leica) in a solution containing (in mM): 10 NaCl, 2.5 KCl, 0.5 CaCl2, 7 $MgSO_4$, 1.25 NaH_2PO_4, 25 $NaHCO_3$, 10 glucose, and 195 sucrose, equilibrated with 95% O2 and 5% CO2. Slices were incubated at 34°C for 30 min in aCSF containing (in mM): 125 NaCl, 2.5 KCl, NaH_2PO_4, $NaHCO_3$, glucose, $CaCl_2$, $MgCl_2$, 2 NaPyruvate. Slices were then maintained at room temperature until they were transferred to a recording chamber on an upright microscope (Olympus) equipped with a 40x objective.

For most whole-cell recordings, electrodes contained (in mM): 130 D-Gluconic acid, 130 CsOH, 5 NaCl, 10 HEPES, 12 phosphocreatine, 3 MgATP, 0.2 NaGTP, 10 EGTA, 0.05 AlexaFluor 594 cadaverine. For experiments measuring miniature IPSCs and TPMPA sensitivity, electrodes contained: 115–125 CsCl, 5 NaCl, 10 HEPES, 10 EGTA, 4 MgATP, 0.3 Na3GTP and 12 phosphocreatine, 0.05 AlexaFluor 594 cadaverine; and NBQX (10 μM) and D-APV (50 μM) were added to the perfusate. Additionally, TTX (1 μM) was added to the perfusate for mIPSC experiments. Voltage-clamp responses were recorded with a Multiclamp 700B amplifier and digitized at 10 kHz (Digidata 1440); evoked responses were low-pass filtered at 4 kHz, and miniature and spontaneous responses were low-pass filtered at 1 kHz. Series resistance was typically ~10 MΩ, always < 20 MΩ, and was compensated at 80%–95%. Data were collected and analyzed offline using AxographX and IGOR Pro (Wavemetrics). Junction potentials were not corrected. Recordings were performed at 35°C. NBQX, D-APV, TTX and gabazine were acquired from Tocris.

Recordings targeted pyramidal cells, which were visualized using a fluorescent indicator (Alexa 594 Cadaverine) to ensure that cells had pyramidal cell morphologies. We recorded in voltage-clamp from uninfected cells adjacent to the infection site. To ensure cells were uninfected we first examined responses to weak, 1 s long light pulses (470 nm, CoolLED) delivered through the 40x objective. Cells that exhibited large and sustained photocurrents were considered ChR2+ and discarded. Uninfected cells were held at either −70 mV or +5 mV to isolate excitatory or inhibitory synaptic currents, respectively. Brief (2 ms, ~10 mW) pulses were delivered every 10 s to activate ChR2+ axon terminals. A concentric bipolar electrode in the lateral olfactory tract was used to activate synaptic inputs from the olfactory bulb.

qEPSCs, uEPSCs and sIPSCs were detected using the automatic Event Detection function in Axograph. Detected events were automatically filtered (amplitude > 8 pA, 10%–90% rise-time < 2 ms) and then manually checked to ensure that all detected events had single rising phases and appropriate decay kinetics. To evoke qEPSCs, the 2 mM $CaCl_2$ in the perfusate was replaced with 2 mM $SrCl_2$. qEPSCs were detected in a window 200-700 ms after the 2 ms light pulse. To evoke uEPSCs, a weak, ramping 5 s long light pulse (0 to ~1 mW) was delivered through a 10x air objective, and events were detected in a window 100-1000 ms after light onset.

mIPSC frequency was calculated by counting the average number of events in a 1-3-minute continuous recording. For each cell, qEPSC, uEPSC and mIPSC amplitudes were determined by averaging > 300 validated events per cell.

Immunohistochemistry

Animals were deeply anesthetized with ketamine/xylazine and perfused through the heart with cold PBS followed by 4% paraformaldehyde 1 hour after the last stimulation of the day. For GABA staining, a 2% paraformaldehyde/2% gluteralderhyde fixative solution was used instead. The optical fibers/optrodes were detached from the skull and the brains were removed and postfixed overnight in 4% paraformaldehyde. Tissue was embedded in a 5% gelatin block and 50 μm-thick coronal sections were cut on a vibrating microtome. To stain for EYFP, cFos, GABA, and parvalbumin, the slices were permeabilized by washing three times with 0.1% PBS-T and were incubated at 4°C overnight in 0.3% PBS-T, goat serum (1:20) and the primary antibodies: chicken anti-GFP (1:500, Abcam ab13970), rabbit anti-cFos (1:500, Cell Signaling Technology 2250S), GABA polyclonal antibody (1:1000, Invitrogen PA5-32241), and parvalbumin polyclonal antibody (1:500, Invitrogen PA1-933). Slices were rinsed and then incubated in goat Alexa 488 anti-chicken (1:500, Abcam ab150169) and Alexa-555 anti-rabbit antibodies (1:500, Invitrogen A27039) and NeuroTrace 640 (1:400, Invitrogen N21483) at 4°C overnight. Staining for GAD2 (rabbit anti-GAD2, 1:500, Cell Signaling Technology 5843S) and somatostatin (rabbit anti-SST, 1:500, Invitrogen 701935) were done on the slices following the same protocol but not included in the analyses due to unquantifiable staining (Figure S6). Following the secondary antibody staining, the slices were thoroughly rinsed and mounted with Vectashield (Vector Laboratories) or Fluoromount-G (Thermo-Fisher, 00-4958-02). All figures were imaged with Zeiss LSM 510 or LSM 780 confocal microscopes and processed by Fiji ImageJ and Adobe Photoshop to uniformly increase brightness and contrast.

Fos, GABA and PV cell counting

GABA-positive neurons were counted within a 500 μm x 500 μm region of interest that was centered on layer 2 and spanned all three PCx layers. Laminar boundaries were determined manually. All sections were counted by two different counters blinded to treatment condition and only used when both counters were in agreement. cFos was quantified using a thresholding method in user-defined areas across layers 2 and 3 in Fiji, followed by the "Multi-point" function, with the counter blind to treatment type. Each count represents the average number of GABA-positive or cFos-positive cells, normalized by area, across four sequential 50 μm slices through either aPCx or pPCx for each mouse.

For the PV measurements, we first used the Fiji "Multi-point" function to identify PV-positive cells in both ipsi- and contralateral pPCx, and then used the "Measure" function to obtain signal intensity measurements for each identified PV cell. Data are presented as the average intensity of ipsilateral PV signal normalized by that of the contralateral hemisphere for each slice (n = 4 slices each from 4 mice for both OpK and sham control groups).

Fluorescent *in situ* hybridization

Mice were transcardially perfused with PBS followed by 4% PFA. The brains were post-fixed in the same fixative overnight at 4°C, cryoprotected in 30% sucrose/PBS overnight at 4°C and embedded in O.C.T. compound. Fluorescent *in situ* hybridization for vesicular GABA transporter (vGat) was performed on 50 μm coronal free-floating sections as described previously [80]. Briefly, sections were hybridized with a digoxigenin-labeled vGat probe overnight at 62°C. Sections were washed, treated with RNase A (20 μg/ml for 45 min at 37°C), and incubated with sheep anti-digoxigenin antibody conjugated with alkaline phosphatase (1:3500; Sigma, 11093274910) and rabbit anti-GFP (1:1000; abcam, ab290). Hybridization was visualized with Fast Red TR/Naphthol AS-MX (Sigma-Aldrich, F4648). Then, sections were incubated with a donkey anti-rabbit secondary antibody conjugated with Alexa Fluor 488 (1:1000; Jackson ImmunoResearch Lab) and DAPI. Images were taken with a confocal microscopy (LSM700; Carl Zeiss).

QUANTIFICATION AND STATISTICAL ANALYSIS

Unless explicitly stated otherwise in the text, all data are given as mean ± standard error. The statistical tests and parameters used to analyze data are detailed in the Results section and in the Figure Legends. Specific qualifications for quantification are delineated in the Method Details under the respective subsections. Statistics were calculated using standard functions in MATLAB and Igor Pro.

Cell Reports, Volume *35*

Supplemental information

Chronic loss of inhibition in piriform cortex following brief, daily optogenetic stimulation

Brendan Ryu, Shivathmihai Nagappan, Fernando Santos-Valencia, Psyche Lee, Erica Rodriguez, Meredith Lackie, Jun Takatoh, and Kevin M. Franks

Supplementary Materials for Ryu et al, 2020

Figure S1. related to Figure 1.

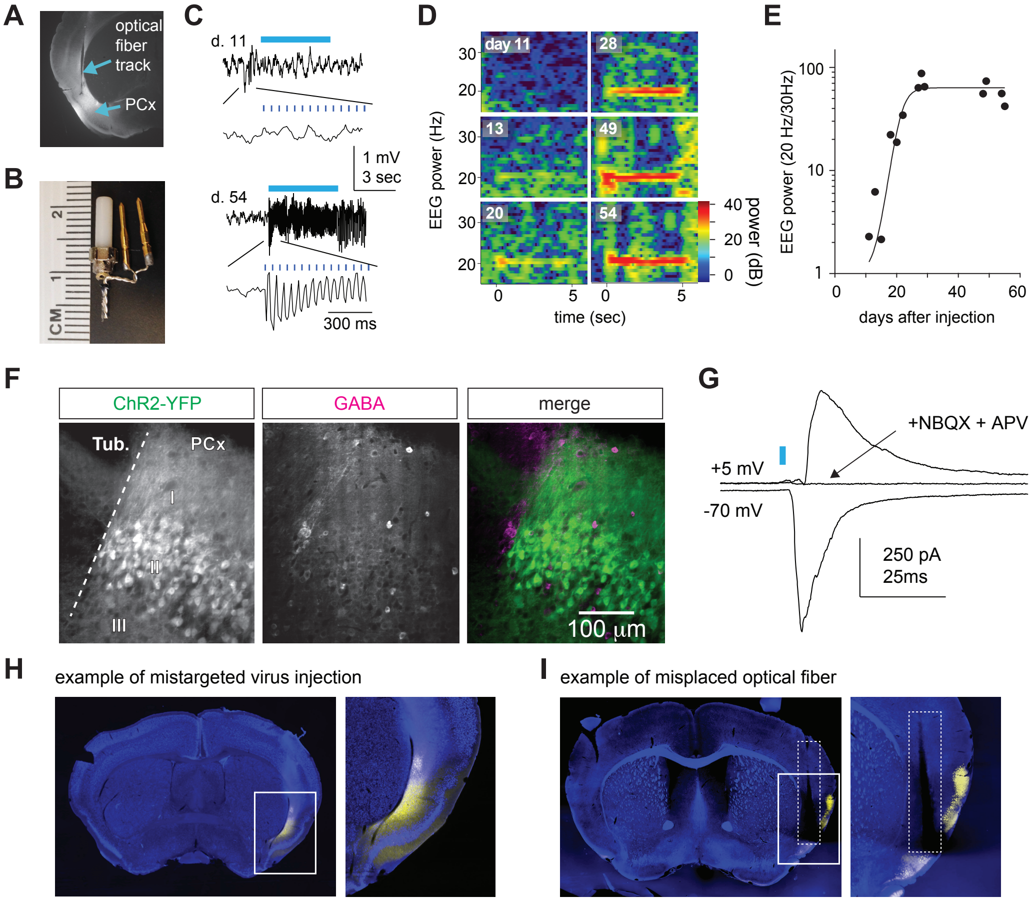

Figure S1. Timeline and specificity of ChR2 expression (**A**) Coronal brain section showing ChR2-YFP expression and track from optical fiber. This animal was implanted with only an optical fiber-coupled ferrule. Tissue damage was considerably more extensive when using optrodes. (**B**) Example optrode. (**C**) Example LFPs in response to brief light pulses at 20 Hz, recorded in the same mouse 11days (*top*) and 54 days (*bottom*) after virus injection. The same light intensity was used for all stimuli. Insets show time of stimulus onset at expanded time scales. (**D**) Spectrograms in response to 20 Hz stimuli at different times after virus injection from the same mouse, including the examples shown in C. (**E**) Evolution of ChR2 expression measured as power of the LFP at 20 Hz normalized by the power of the LFP at 30 Hz during stimulation. As ChR2 expression increases the LFP becomes increasingly stimulus-locked until it plateaus 3 weeks after virus injection. Solid line is a sigmoidal fit to the data. (**F**) ChR2-YFP expression at the boundary of the PCx, in which most cells are glutamatergic, and the olfactory tubercle, which is predominantly GABAergic. Note the abrupt termination of ChR2-YFP expression at the PCx-tubercle boundary. Note also that the few GABA-positive neurons in PCx do not express ChR2-YFP. Scale bar is 100 µm. (**G**) Example whole-cell voltage-clamp recording from an uninfected neuron in response to a brief (2 ms) light pulse (blue bar). Light evoked strong inward currents (i.e. EPSCs) at V_m = -70 mV and outward currents (IPSCs) at V_m = +5 mV. IPSCs were completely blocked by glutamate receptor antagonists (NBQX, 10 µM; D-APV 50 µM), consistent with disynaptic feedback

inhibition with ChR2 expression being selective for glutamatergic and not GABAergic neurons. IPSCs were also blocked by gabazine (10 µM, not shown). (**H**) Example of an experiment in which virus injection was mistargeted so that there was negligible ChR2-YFP expression in PCx. Panel on right shows expanded region in box on left. (**I**) Example of an experiment in the optical fiber was lowered through PCx. Panel on right shows expanded region in box on left. Dashed line demarcates fiber tract.

Figure S2. related to Figure 1.

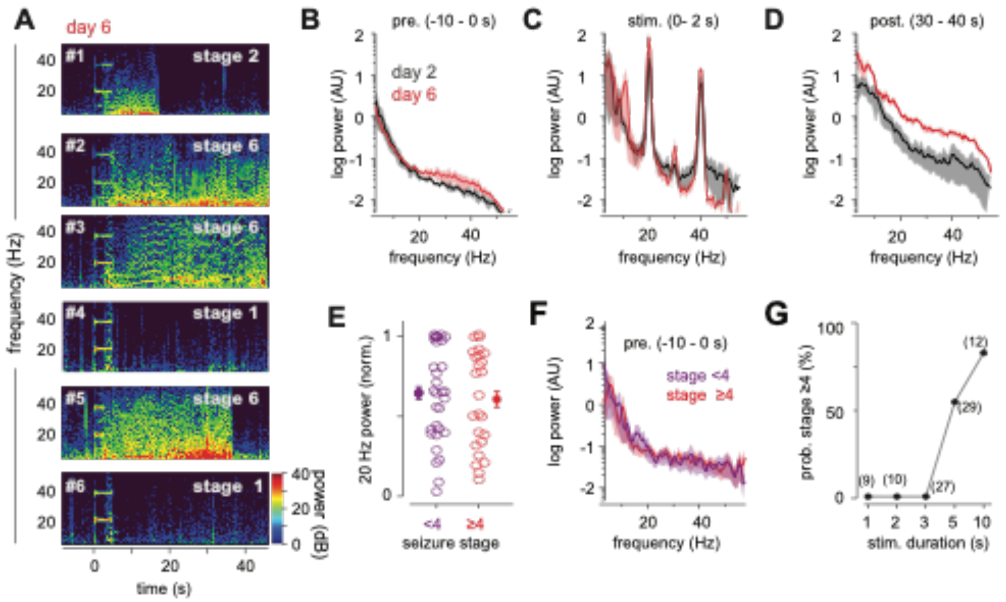

Figure S2. Strong seizures are only evoked on a subset of trials. (**A**) Example spectrograms for all stimuli on day 6 from a representative OpK animal. Stimulus number is indicated in top left. Note that stimuli 1, 4 and 6 fail to evoke seizures. (**B**) Average LFP power spectra during the 10 s before stimulus onset, averaged across all trials from animals with implanted electrodes (n = 5 mice) on day 2 (black trace) and day 6 (red trace). (**C**) As in B, but for the first 2 s of the 20 Hz stimulus train. (**D**) As in B, but for 10 s. starting 25 s after the stimulus ended. (**E**) The was no difference in stimulus strength on trials that did or did not evoke seizures in OpK mice. Graph shows power of LFPs at 20 Hz during first 2 s of all 6 stimuli on days 5 and 6. Trials are sorted by stimuli that unsuccessfully (purple circles) or successfully (red circles) evoked a stage ≥4 seizure on each day, and normalized by the maximum power at 20 Hz recorded on each day, regardless of whether that trial was successful (mean ± s.e.m.: seizure trials, 0.65 ± 0.053, n = 32 trials from 5 mice; no seizure trials, 0.61 ± 0.061, n = 25 trials from 5 mice; p = 0.53, Mann-Whitney U test). (**F**) Average power spectra of LFPs before stimulus onset for all trials on days 5 and 6 sorted by trials that did (red trace) and did not (purple trace) evoke stage ≥4 seizures. We found no obvious differences in LFPs before stimulation in trials that did and did not evoke seizures. (**G**) Probability of evoking a stage ≥4 seizure in already optokindled mice (i.e. beyond the first 6 days) as a function of stimulation duration. Each day a single 5 s stimulus was presented first and was not included in this analysis. Numbers above each point indicate number of trials for each duration.

Figure S3, related to Figure 1.

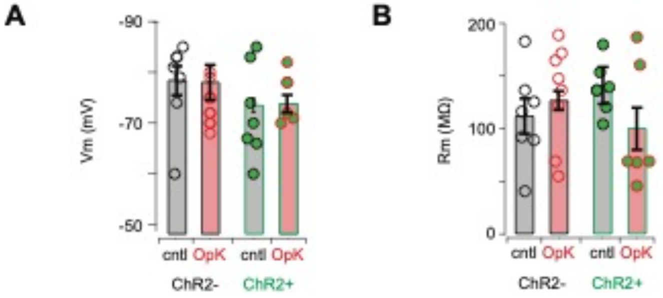

Figure S3. No change in intrinsic excitability or EPSC paired-pulse ratios. (**A**) Resting membrane potentials (V_m) were equivalent in ChR2-positive (green outlines) and uninfected cells (black outlines) from both OpK (red shading) or control (grey shading) animals. Two-way ANOVA found no significant effects for ChR2 expression ($F_{1,24}$=.0.447, p=0.510), optokindling ($F_{1,24}$=.0.121, p=0.731), or an interaction $F_{1,24}$=.2.16, p=0.154). (**B**) As in A, but for input resistance. Two-way ANOVA found no significant effects for ChR2 expression ($F_{1,24}$=.0.242, p=0.627), optokindling ($F_{1,24}$=.0.036, p=0.852), or an interaction $F_{1,24}$=.2.94, p=0.010).

Figure S4, related to Figure 3.

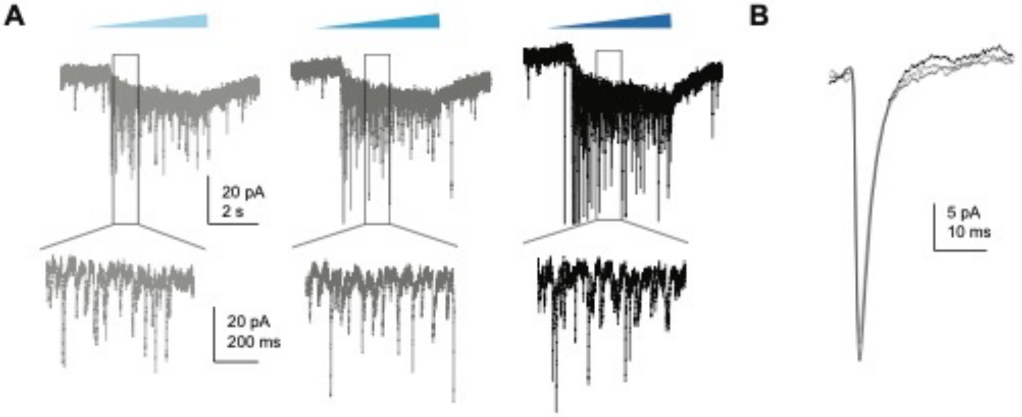

Figure S4. Light intensity changes uEPSC frequency but not amplitude. (**A**) Three traces from an example recording in which uEPSCs were evoked by ramping 5-second light pulses at increasing intensities (blue triangels). Traces below correspond to insets indicated by the boxes on traces above. (**B**) Overlaid uEPSCs averaged over at least 100 well-isolated individual events recorded at each of the three stimulus intensities.

Figure S5, related to Figure 5

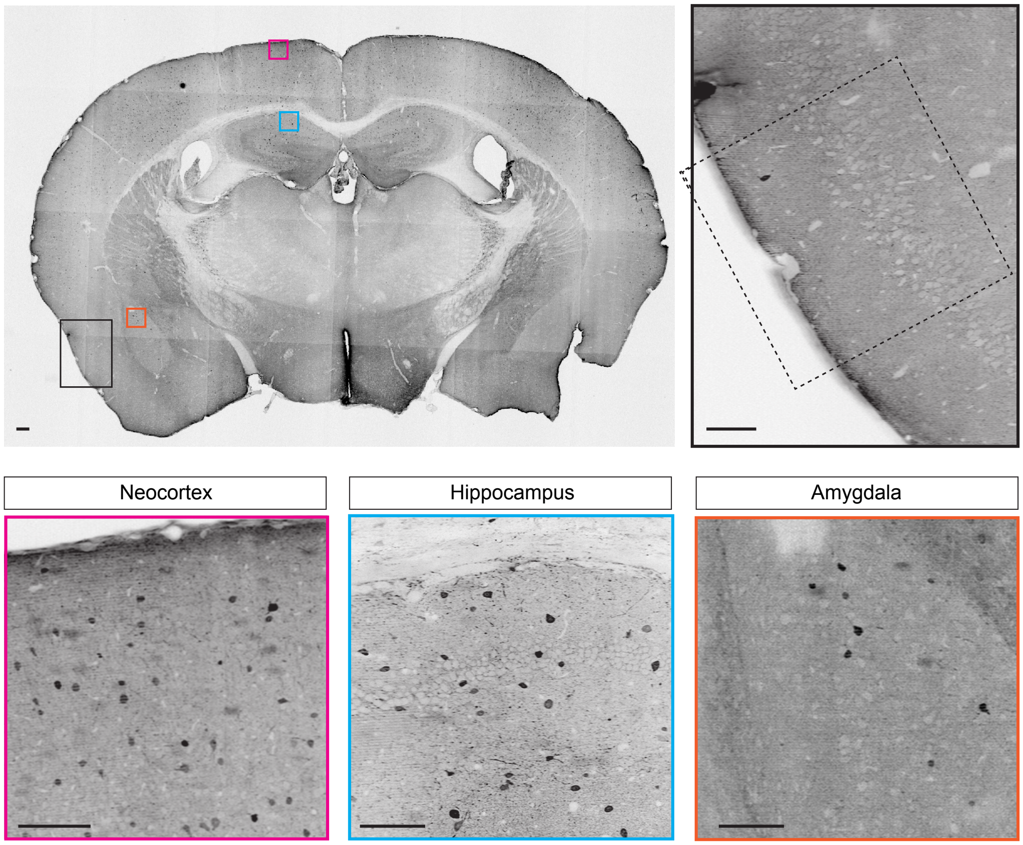

Figure S5. Robust GABA expression outside of PCx.
Coronal section from an OpK mouse stained for GABA. Insets show enlarged sections from PCx (*right*), and ipsilateral neocortex, hippocampus and amygdala. Note that GABA staining is depleted in PCx but robust elsewhere. Dashed box over PCx insert indicates the 500 µm x 500 µm window that was aligned to the top of layer I and used for quantification. All scale bars are 100 µm. All sections were harvested 24 hours after the sixth purple trace stimulation.

Figure S6, related to Figure 6.

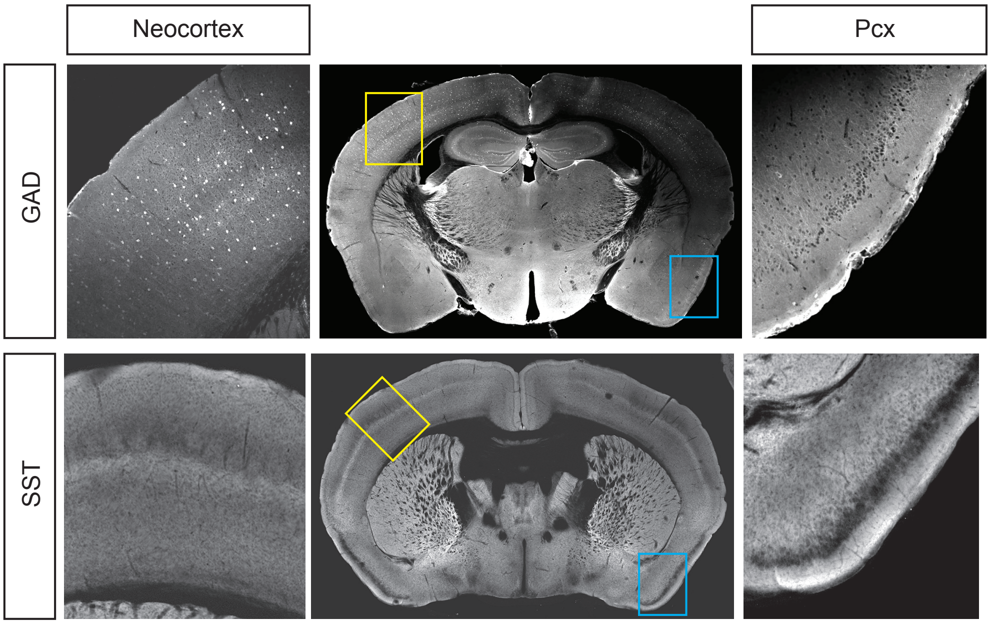

Figure S6. Failed labeling of PCx interneurons with GAD and SSt antibodies.
Example of whole-brain coronal sections stained using either GAD (*top*, rabbit anti-GAD2, 1:500, Cell Signaling Technology 5843S) or SSt antibodies (*bottom*, rabbit anti-SST, 1:500, Invitrogen 701935). In all cases we failed to label interneuron somata in PCx. Panels on left and right show magnified images of the neocortex (yellow box) and pPCx (blue box) on the main panel. Note, for example, that we could clearly label neocortical GAD-positive neurons in neocortex but not in pPCx in the same section.
